# Experimental Investigation into the Reactions Between Liquid Gallium and Inorganic Nitrogen Precursors (N_2_, NH_3_, and NH_4_Cl) at 400–500 °C

**DOI:** 10.3390/ma19101955

**Published:** 2026-05-09

**Authors:** Yuxi Zheng, Xiaofei Guan

**Affiliations:** School of Physical Science and Technology, ShanghaiTech University, 393 Huaxia Middle Road, Shanghai 201210, China; zhengyx2023@shanghaitech.edu.cn

**Keywords:** liquid gallium, nitridation, gallium nitride, interfacial reaction, oxide layer

## Abstract

Liquid gallium (Ga) provides a dynamic reaction interface covered by a self-limiting native oxide layer, yet the reaction behavior of liquid Ga with different inorganic nitrogen sources and the surface-layer evolution remains insufficiently clarified. Herein, we have comparatively investigated interfacial reactions of pure liquid gallium (Ga) with N_2_, NH_3_, and NH_4_Cl under controlled thermal treatments (400, 450, or 500 °C for a 6 h duration), and further examined the reaction with NH_4_Cl in non-contact versus direct-contact configurations. The resulting surface films were analyzed using a combination of multiple characterization tools after removing residual liquid Ga underneath. Under N_2_ at 400–500 °C, the surface products obtained were dominated by oxygen-containing gallium species and no distinguishable nitride phase was detected, indicating sluggish kinetics of nitridation in this temperature range. In comparison, NH_3_ promoted nitrogen incorporation more effectively. Nitrogen-related signals were also detected in the surface products of the NH_4_Cl experiments in non-contact and direct-contact modes, whereas direct contact resulted in significantly stronger interfacial restructuring and characteristic morphologies, such as spheres and hollow-shell structures. Overall, the extent of nitrogen incorporation and the morphology evolution are jointly governed by nitrogen-source reactivity, temperature, and local contact conditions, with the native oxide layer mediating the competing oxidation and nitridation processes.

## 1. Introduction

The formation of the Ga–N bond is directly linked to the synthesis of gallium nitride, a wide-bandgap semiconductor that is widely used in power electronics, radio frequency devices, and solid-state lighting [1,2,3,4]. Currently, the primary industrial processes for synthesizing gallium nitride include Metal–Organic Chemical Vapor Deposition (MOCVD), Hydride Vapor Phase Epitaxy (HVPE), and the ammonothermal method [5,6]. In MOCVD, gallium metal organics (e.g., trimethyl gallium) and ammonia gas are introduced into a reactor, where they undergo thermal decomposition on the surface of a heated substrate to deposit a gallium nitride film [7,8]. In HVPE, metallic gallium reacts with hydrogen chloride gas to form gaseous gallium chloride, which is then transported to the substrate region to react with ammonia, resulting in the growth of gallium nitride crystals [9]. In ammonia–thermal method, GaN feedstock dissolves in supercritical ammonia containing mineralizers, is transported by convection, and then crystallizes on native seeds in the supersaturated growth zone [10]. Significant progress on industrial process optimization has been achieved so far [11]. In addition, researchers have focused on the activation of N_2_ with various approaches for GaN synthesis at milder temperatures. For example, Imaoka et al. have reported using a plasma-based neutral beam method to generate high-kinetic-energy N_2_ molecules to react with GaCl_3_ on different substrates, thereby achieving the synthesis of GaN at room temperature [12].

Furthermore, the interaction between liquid gallium and nitrogen gas is closely related to the synthesis of ammonia, a key feedstock for nitrogen fertilizer production. The ammonia synthesis process currently used in industry (the Haber-Bosch process) is highly energy-intensive due to harsh reaction conditions (400–500 °C and 150–250 bar) and requires large-scale centralized operation to enable economic viability [13,14]. To develop efficient catalysts for distributed ammonia synthesis at milder conditions, Zuraiqi et al. utilized a 2wt%Cu–98wt%Ga catalyst to achieve ammonia synthesis at 400 °C and 1 to 4 bar, with the catalyst’s performance rivaling that of Ru-based catalysts [15]. This study introduced transition metals (e.g., Cu) into liquid gallium and dissolved them, opening up a new dimension for the design of ammonia synthesis catalysts [16]. Wang et al. reported ammonia synthesis using solid LiGa intermetallic compound at 400 °C and 1 bar [17]. In addition, liquid alloys comprising reactive metals (e.g., Li, Mg, Ca) and low-melting-point solvent metals (e.g., Sn, Pb, Zn) similar to Ga have also been previously investigated for nitrogen fixation and ammonia synthesis in various systems [13,18,19,20,21]. Given the above progress, further research into the Ga–N bond formation will be of vital importance for advancing the development of Ga-based catalysts for sustainable ammonia production.

Although previous studies have accumulated a wealth of knowledge regarding GaN growth and Ga-based catalytic ammonia synthesis, a key challenge remains: Ga is difficult to nitride, and it tends to form an oxide layer more readily instead. In actual reactions, oxidation often competes with nitridation. This is because of the high N≡N bond energy of nitrogen molecules (942 kJ mol^−1^) and relatively low bond energy of oxygen molecules (495 kJ mol^−1^); in addition, the energy required to generate nitrogen anions (2300 kJ mol^−1^ for N^3−^) is much greater than that required to generate oxygen anions (700 kJ mol^−1^ for O^2−^) [12].

Overall, a systematic investigation into the relative ease of nitridation using different nitrogen sources is not quite sufficient, especially in the 400–500 °C temperature range, although substantial progress has been made in nitridation-related studies [22,23,24,25]. Based on this, this paper selects three representative inorganic nitrogen sources—N_2_, NH_3_, and NH_4_Cl—to systematically investigate their reaction behavior with liquid Ga and further compare the NH_4_Cl experiments under non-contact and direct-contact conditions. By combining characterization techniques such as X-ray Diffraction (XRD), X-ray Photoelectron Spectroscopy (XPS), Scanning Electron Microscopy/Energy-dispersive X-ray Spectroscopy (SEM/EDS), and Transmission Electron Microscopy (TEM), we analyze the phase composition, chemical state, and morphological evolution of products formed on the liquid Ga surface. This enables us to evaluate the ease of nitriding liquid Ga with different nitrogen sources and further elucidate the effects of the native oxide layer, temperature, and contact mode on the formation of Ga–N bonds at the liquid gallium interface and the evolution of the resulting products.

## 2. Materials and Methods

### 2.1. Materials and Gas Supply

Gallium (Ga; Titan^TM^, Shanghai, China, 99.99% purity, 2.0 g ± 1%) and ammonium chloride (NH_4_Cl; Titan^TM^, >99.9%, 1.0 g ± 1%) were used as received. Ultra-high-purity N_2_ (Weichuang^TM^, Shanghai, China; 99.999%) was used as a nitrogen source. Ammonia was supplied as a premixed cylinder gas (5% NH_3_–95% Ar, Weichuang^TM^, 99.999%). Ultra-high-purity Ar (Weichuang^TM^, 99.999%) was used in the NH_4_Cl experiments.

### 2.2. Reactor Setup and Protocols

#### 2.2.1. Preparation of Clean Pristine Liquid Ga

Commercial Ga granules were handled in an Ar-filled glovebox (Vigor^TM^, Suzhou, China) to minimize oxidation prior to experiments. The Ga granules were transferred to a low-borosilicate glass flask, which was placed on a hot plate (HSC-19T, JOANLAB^TM^, Huzhou, China). The temperature was set to 60 °C to melt the Ga. Because the as-received Ga granules carried a native oxide layer, a syringe was used to withdraw Ga from the interior of the liquid. The clean liquid Ga obtained was then transferred into a new glass vial, capped and stored inside the glovebox. Before each experiment, the stored gallium was reheated to 60 °C and dispensed with a syringe for sample loading.

#### 2.2.2. Control of Oxygen Content in the Feeding Gas

The ultra-high-purity N_2_ or Ar commercially purchased typically contains 1–10 ppm of oxygen-containing species (e.g., O_2_, H_2_O). Before entering the reactor, the oxygen content of the gas was reduced using a bubbling method (Figure S1). A liquid Ga column (height: 10 cm) was used as the oxygen scavenger due to its high reactivity. During operation, the tube furnace heated the liquid Ga to 200 °C when NH_3_ was used, or to 400 °C when N_2_ or Ar was used. Although these measures reduced the oxygen background, trace amounts of oxygen still inevitably entered the reactor because of limitations in the practical experimental setup and gas purity. Note that a flexible PTFE tube with an outer diameter of 6 mm, an inner diameter of 4 mm and a length of ~400 mm was used to connect the stainless-steel gas line to the reactor. Oxygen ingress or permeation through such practical tubing and fittings could not be completely excluded. Therefore, oxidation remained an important competing process under the present conditions, and the oxygen level was not rigorously quantified in situ during reaction. In future work, a zirconia-based oxygen sensor can be used to determine the oxygen partial pressure in the test gas [26].

#### 2.2.3. Reactions with N_2_ or NH_3_

**Figure 1:**
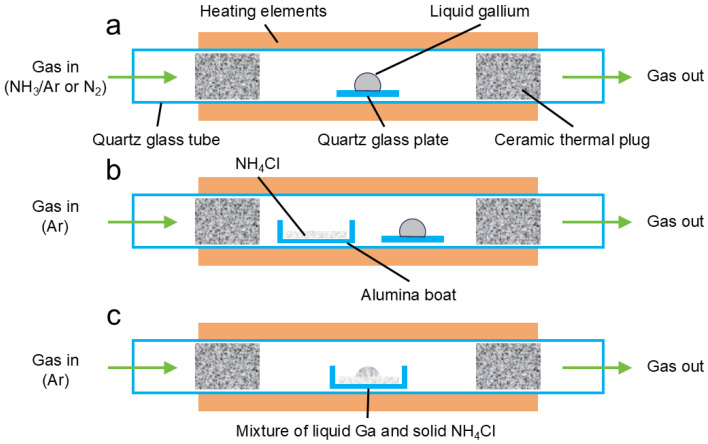
Schematics of the reactors for investigating (**a**) the reaction between liquid Ga and N_2_ or 5%NH_3_–95%Ar and (**b**,**c**) the reaction between liquid Ga and solid NH_4_Cl in either (**b**) non-contact mode or (**c**) direct-contact mode. In (**a**), liquid Ga was supported on a quartz glass plate. In (**b**), NH_4_Cl was placed in an alumina crucible and Ga was supported on a quartz glass plate. In (**c**), NH_4_Cl was mixed with liquid Ga in an alumina crucible.

Reactions with nitrogen sources were performed in a horizontal single-zone tube furnace (BTF-1200C-S, BEQ^TM^, Hefei, China) equipped with a quartz glass tube (Figure 1a). Samples were positioned at the center of the furnace hot zone to ensure a stable and reproducible thermal environment. For N_2_ and 5% NH_3_/Ar experiments, liquid Ga (2.0 g ± 1%) was loaded onto a quartz glass plate (25 mm × 25 mm; Donghai Yibo^TM^ Quartz, Lianyungang, China) as the support substrate. All sample loading and reactor assembly steps were conducted inside an Ar-filled glovebox. Afterward, the reactor sealed with vacuum flanges and parafilm was transferred out of the glovebox and immediately connected to the gas lines, during which a small quantity of air might be introduced to the reactor. Then, the reactor was purged for 8 h using the reaction gas at room temperature. The 8 h purging time was determined from the GC experiment (Note S1). After purging, the reactor was then heated at 5 °C·min^−1^ to the target temperature (400, 450, or 500 °C), held for 6 h, and then cooled to room temperature. The feeding gas was switched to Ar during cooling. The total flow rate was always 20 mL·min^−1^ at ambient pressure, as controlled with mass flow controllers (Omega Engineering^TM^, Norwalk, CT, USA). In addition to the 400–500 °C experiments, a selected higher-temperature N_2_ comparison experiment was performed at 800 °C for 6 h under otherwise similar gas-flow and heating conditions, in order to evaluate whether stronger thermal activation could promote N_2_-derived nitridation.

#### 2.2.4. Reactions with NH_4_Cl in Non-Contact and Direct-Contact Modes

For NH_4_Cl-assisted experiments, liquid Ga was loaded into alumina crucibles (Yixing Purshee^TM^, Yixing, China) to accommodate the solid nitrogen source and to enable defined non-contact and direct-contact configurations. In these experiments, NH_4_Cl powders (1.0 g ± 1%) were used. In the non-contact configuration, NH_4_Cl and Ga were placed in two separate alumina crucibles within the same heated zone. The NH_4_Cl-containing crucible was positioned on the upstream side near the gas inlet, whereas the Ga-containing quartz glass plate was placed near the center of the hot zone. This arrangement prevented direct physical contact and allowed Ga to interact only with the gas-phase species released from NH_4_Cl (Figure 1b). In the direct-contact configuration, liquid Ga and NH_4_Cl were first placed in the same 60 mL HDPE bottle (Chengdu Huangyu^TM^, Chengdu, China) under Ar protection and vigorously mixed using a high-speed shaker before being transferred into a single alumina crucible, thereby enabling both gas-phase exposure and direct interfacial interaction (Figure 1c). For both configurations, the reactor was purged with Ar for 8 h prior to heating, and the reaction was carried out under Ar at 20 mL·min^−1^. The temperature program was identical to that used for the gaseous nitrogen-source experiments. The experimental details, including reaction conditions, are summarized in Table 1.

### 2.3. Characterization

#### 2.3.1. XRD Characterization

Phase identification and crystal structure analysis were carried out using a Bruker D8 Advance powder X-ray diffractometer equipped (Bruker, Beijing, China) with a Cu K*α* radiation source in *θ*–2*θ* geometry. The instrument was operated at 40 kV and 40 mA. Diffraction patterns were collected over a 2*θ* range of 10–80° with a step size of 0.02° and a scan rate of 0.2 s per step. Phase identification was performed by comparison with the ICDD PDF-4+ database. In Exp. C1, Exp. A1, and Exp. S1 (Table 1), each film sample was obtained by withdrawing the liquid Ga metal underneath using a syringe, and then a deep-grooved acrylic sample holder with modeling clay was used to support the entire quartz glass and the film sample, and then a clean microscope slide was used to press the peripheral region of the quartz glass until it was flush with the acrylic sample holder. In some cases, a monocrystalline silicon sample holder was also used for film samples. For the powder sample from Exp. S2 (Table 1), ~50 mg was weighed out and placed on a monocrystalline silicon sample holder, then flattened using a clean glass slide.

#### 2.3.2. SEM/EDS Characterization

The surface morphology of the reaction products was examined using a JSM-7800F (JEOL^TM^, Musashino, Japan) scanning electron microscope, and elemental distribution was analyzed using the attached energy-dispersive X-ray spectroscopy (EDS) detector. For sample preparation, conductive carbon tape was first attached to a sample stub, after which a small amount of sample was transferred onto the tape with tweezers and gently purged with an air gun. For secondary-electron imaging and EDS elemental mapping, the microscope was operated at an accelerating voltage of 5 kV, a working distance of 7 mm, and a probe current setting of 6 nA. The imaging parameters were further adjusted when necessary to obtain clear micrographs for different samples.

#### 2.3.3. TEM Characterization

Transmission electron microscopy (TEM) observations were conducted on a JEM-2100 Plus instrument (JEOL^TM^, Musashino, Japan) to characterize the atomic structure of the products obtained from the reactions of liquid Ga. For TEM sample preparation, a small amount of sample was dispersed in ethanol and ultrasonicated to obtain a uniform suspension. Subsequently, 100 μL of the suspension was deposited dropwise onto the front side of a 200-mesh copper grid and dried thoroughly in a fume hood before observation.

Aberration-corrected TEM and STEM-EDS analyses were further performed on a JEOL GrandARM300F instrument (JEOL^TM^, Musashino, Japan) equipped with a cold field-emission gun, TEM/STEM spherical aberration correctors, and dual JEOL SDD EDS detectors. The EDS energy resolution was 133 eV at Mn Kα. This instrument was used for the site-specific characterization of the FIB-prepared 500 °C non-contact NH_4_Cl sample shown in Figure S42.

#### 2.3.4. FIB Sample Preparation and Evaluation

Focused ion beam (FIB) operation was performed using a JIB-4700F (JEOL^TM^, Musashino, Japan) system equipped with a Ga^+^ ion source. The FIB accelerating voltage range was 1–30 kV, and the probe current range was 1 pA–90 nA. For the representative 500 °C non-contact NH_4_Cl sample, FIB was used to prepare the specimen for aberration-corrected TEM and STEM-EDS analyses on the GrandARM300F instrument described in Section 2.3.3. The final thickness of the FIB-prepared lamella was approximately 70–80 nm. This site-specific preparation enabled local structural and elemental characterization of a selected microstructure formed in the non-contact NH_4_Cl system.

For the 500 °C direct-contact NH_4_Cl sample, FIB-based preparation of cross-sectional TEM specimens was also attempted. During this attempt, ion-beam focusing was performed at an accelerating voltage of 30 kV with a high-current setting of 30,000 pA (30 nA). SEM images were recorded before and after ion-beam focusing to evaluate the morphology stability of the product under FIB irradiation. A pronounced morphology change was observed after ion-beam exposure, indicating that the loosely aggregated/shell-like products in the direct-contact sample were sensitive to high-current ion-beam irradiation. Therefore, FIB-based preparation of representative cross-sectional TEM specimens from this sample was considered unreliable under the present operating conditions.

#### 2.3.5. XPS Characterization

Surface chemical states were analyzed by X-ray photoelectron spectroscopy (XPS) using a Thermo Fisher Scientific ESCALAB^TM^ 250Xi (ESCALAB^TM^, Waltham, MA, USA) spectrometer equipped with an Al K*α* X-ray source (1486.6 eV). To preserve the as-reacted surface and minimize ambient exposure, the samples were prepared in an Ar glovebox and then transferred to the XPS instrument using a portable sample transfer bin (Fermi^TM^ Instruments, Shanghai, China) filled with Ar. During preparation, each sample was placed on a double-sided adhesive tape (Scotch^TM^ 665, 3M, St. Paul, MN, USA) attached to the sample holder. For the pristine Ga sample, the Ga droplet was additionally pressed with a clean glass slide. The XPS analysis spot size was 500 μm, and charge compensation was enabled during data acquisition.

Survey spectra were collected at a pass energy of 50 eV with an energy step of 1.0 eV. High-resolution spectra were acquired at a pass energy of 30 eV with an energy step of 0.1 eV and a dwell time of 50 ms. For high-resolution scans, 5 sweeps were used for most core levels, while 10 sweeps were used for Ga 3*d* and N 1*s* to improve the signal-to-noise ratio. High-resolution spectra of Ga 3*d*, N 1*s*, and O 1*s* were collected for all samples. Binding energies were calibrated against the adventitious C 1*s* peak at 284.8 eV, and elemental valence states were assigned with reference to the NIST XPS database. Because the N 1*s* region overlaps with the Ga LMM Auger feature, assignments in this region were made cautiously and were not based solely on the feature near ~397 eV. Instead, the N 1*s*/Ga LMM window was interpreted by comparison with pristine Ga and by cross-checking the Ga 3*d*, Ga 2*p*, O 1*s*, and survey spectra. Due to the strong spectral overlap and the possible chemical-state dependence of the Ga LMM Auger profile, quantitative deconvolution of this region was not attempted.

## 3. Results and Discussion

### 3.1. Thermodynamic Calculations and Analysis of Ga Reactions with Various Chemicals

Ga(l) + 3/4O_2_(g) = 1/2Ga_2_O_3_(s) (1)

Ga(l) + 1/2N_2_(g) = GaN(s) (2)

Ga(l) + NH_3_(g) = GaN(s) + 3/2H_2_(g) (3)

Ga(l) + NH_4_Cl(s) = GaN(s) + 3/2H_2_(g) + HCl(g) (4)

**Figure 2:**
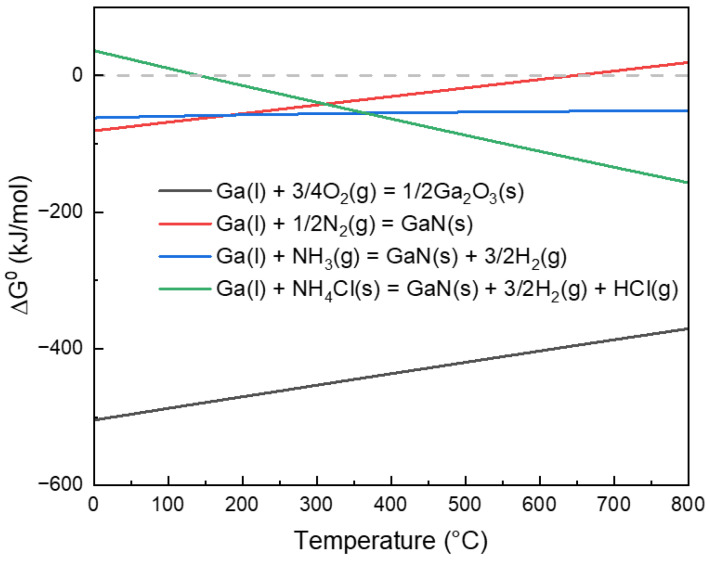
Temperature dependence of the standard Gibbs free energy change (ΔG^0^) for the reactions of liquid Ga with O_2_, N_2_, NH_3_, and NH_4_Cl. The data were obtained from HSC 5.1 Chemistry Database.

Figure 2 shows the temperature dependence of the standard Gibbs free energy changes for the reactions of liquid Ga with O_2_, N_2_, NH_3_, and NH_4_Cl. In the range of 0–800 °C, the ΔG^0^ for Equation (1) is consistently the most negative, indicating that the oxidation of Ga to form Ga_2_O_3_ has the strongest thermodynamic driving force. Therefore, under oxygen-containing conditions, liquid Ga preferentially undergoes oxidation, and the formation of Ga_2_O_3_ will significantly compete with the formation of GaN. In contrast, the reaction curves for Equations (2) and (3) lie below the zero line throughout the entire temperature range under consideration, indicating that both pathways are thermodynamically feasible for GaN formation. However, the ΔG^0^ of the former increases significantly with rising temperature, indicating that heating weakens the thermodynamic driving force for the direct reaction between Ga and N_2_; the ΔG^0^ of the latter, on the other hand, changes little with temperature, suggesting that the nitridation process involving NH_3_ exhibits relatively stable thermodynamic feasibility within this temperature range. For Equation (4), its ΔG^0^ is positive in the low-temperature region, indicating that the reaction is thermodynamically unfavorable at low temperatures; as the temperature increases, ΔG^0^ continuously decreases and turns negative near approximately 130 °C, suggesting that heating significantly favors the reaction, and above approximately 400 °C, its thermodynamic driving force further exceeds that of the N_2_ and NH_3_ nitridation pathways.

In summary, across the entire temperature range studied, the oxidation of Ga is the most favorable reaction. The thermodynamic driving force for direct nitridation via N_2_ significantly weakens with increasing temperature. The nitridation via NH_3_ maintains relatively stable thermodynamic feasibility. In contrast, the reactions involving NH_4_Cl exhibit a more pronounced thermodynamic preference at higher temperatures. Note that the above analysis is based on standard-state ΔG^0^, which primarily reflects the thermodynamic trends of the reactions; the actual reaction process will still be influenced by the combined effects of gas partial pressures, interfacial conditions, and kinetic factors.

### 3.2. Characterization of the Surface of As-Prepared Pure Liquid Ga

**Figure 3:**
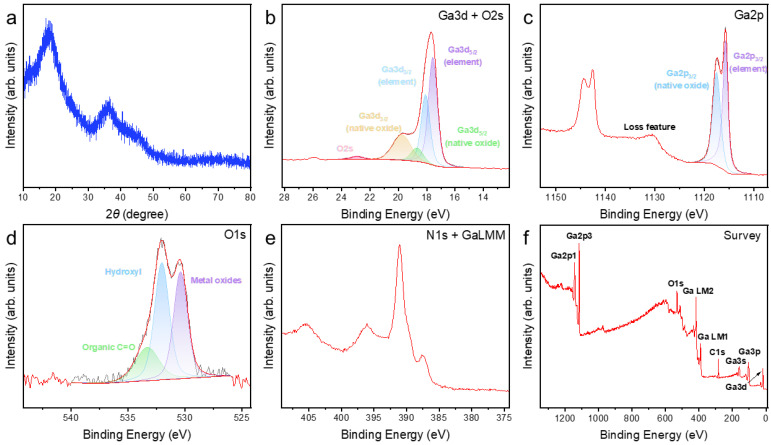
Characterization Results of Pristine Liquid Ga. (**a**) XRD pattern; XPS characterization: (**b**) Ga 3*d* + O 2*s* high-resolution XPS result; (**c**) Ga 2*p* high-resolution XPS result; (**d**) O 1*s* high-resolution XPS result; (**e**) N 1*s* + Ga LMM high-resolution XPS result; (**f**) Survey scan.

The pristine liquid gallium exhibited no obvious crystalline diffraction features at room temperature (Figure 3a). The broad peak at 2*θ* ≈ 18° was attributed to the amorphous scattering halo of the surface polyimide coating layer (Figure S2), whereas a broad peak and a shoulder centered at 2*θ* ≈ 35° and 2*θ* ≈ 45°, respectively, arose from the contribution of the short-range ordered structure of liquid gallium [27]. The XPS results of both Ga 3*d* (Figure 3b) and Ga 2*p* (Figure 3c) exhibited signals corresponding to elemental gallium and its native oxide on the analyzed surface. Note that XPS is renowned for its extreme surface sensitivity, typically probing only the top 10 nm or less of a material [28]. In addition, the O 1*s* spectrum (Figure 3d) indicated the presence of surface oxidation as well as partial organic contamination on the sample. It is particularly noteworthy that the N 1*s* signal and the Ga LMM Auger signal typically exhibit overlap [1,29]. Based on the peak profile, database comparison, and the absence of other nitrogen-related evidence, the signal near 397 eV in the pristine Ga sample was assigned mainly to the Ga LMM Auger feature rather than to N 1*s*. This spectrum was therefore used as an important reference for interpreting the N 1*s*/Ga LMM region of the reacted samples. Raman characterization revealed no significant features of crystallized surface oxides [30] (Figure S3). These results collectively suggested that the as-prepared liquid gallium surface was covered primarily by an ultrathin native oxide layer [31,32,33].

This initial surface state should be central to the subsequent reaction behavior with various nitrogen sources. The presence of native oxide layer implied that the actual reaction interface was not a fully exposed metallic gallium surface, but rather a chemically heterogeneous interface involving oxide-covered regions and locally exposed metallic gallium beneath or around the native skin [33,34,35]. Therefore, any interpretation of the nitridation behavior of liquid Ga must consider the oxide layer as an initial condition rather than a negligible background feature.

### 3.3. Reaction Behavior Under N_2_

#### 3.3.1. Macroscopic Evolution and Phase Identification

**Figure 4:**
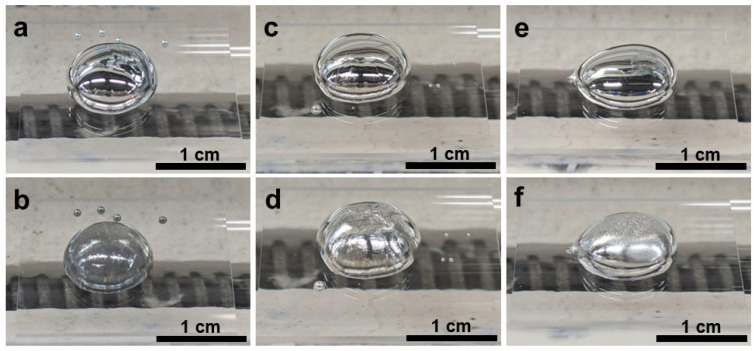
Digital photographs taken at room temperature of liquid Ga samples on quartz glass before reaction at (**a**) 400 °C, (**c**) 450 °C, and (**e**) 500 °C and after reaction at (**b**) 400 °C, (**d**) 450 °C, and (**f**) 500 °C in N_2_ atmosphere.

After thermal treatment under N_2_ at 400–500 °C, the initially mirror-like gallium droplets (Figure 4a,c,e) slightly lost their metallic gloss (Figure 4b,d,f) and developed increasingly roughened and wrinkled surfaces with increasing temperature. The macroscopic transition from a reflective fluid surface to a matte, textured surface suggested the formation of a solid reaction layer (Figure 4).

**Figure 5:**
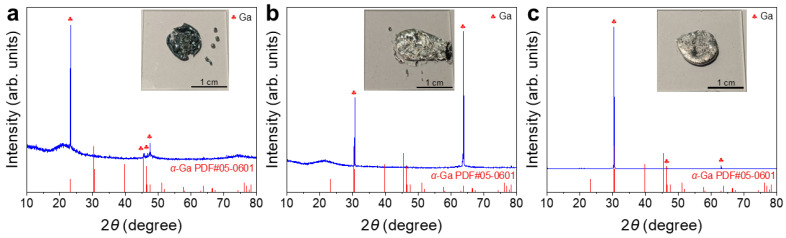
XRD patterns of liquid Ga surface films after heat treatments in N_2_ at (**a**) 400 °C, (**b**) 450 °C, and (**c**) 500 °C. Insets are digital photographs of the post-experimental samples with the majority of liquid Ga underneath withdrawn.

Surface films were obtained by withdrawing the liquid Ga underneath. XRD patterns of the films showed some of the characteristic peaks of *α*–Ga. The solidified Ga in the film preferentially exhibited certain crystallographic orientations under the influence of the top surface layer or the quartz glass substrate [36]. The broad diffraction peak at ~22° (Figure 5a,b) was attributed to the quartz glass substrate (Figure S2). No distinguishable nitride phase was detected over the whole temperature range (Figure 5), indicating the great difficulty of liquid Ga nitridation by N_2_.

#### 3.3.2. Morphology and Chemical States

**Figure 6:**
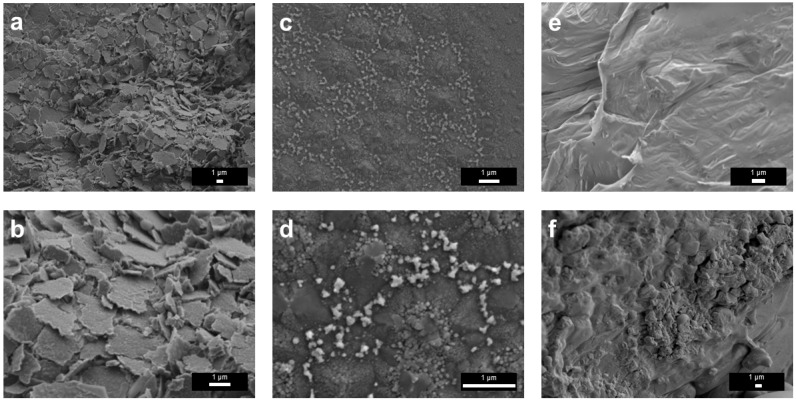
SEM results of liquid Ga films after heat treatment in N_2_ atmosphere at different temperatures. (**a**,**b**) 400 °C; (**c**,**d**) 450 °C; (**e**,**f**) 500 °C.

SEM characterization was conducted to examine the surface films’ morphology. After the treatment at 400 °C in N_2_, the film surface consisted of stacked flaky and lamellar fragments, indicating the formation of a fragile solid layer (Figure 6a,b). For the sample treated at 450 °C, the surface was relatively more continuous and was decorated with fine particle-like or island-like features (Figure 6c,d). In addition, some of the brighter spots in Figure 6d were Ga that escaped through cracks on the surface film based on the EDS results (Figure S5). For the sample treated at 500 °C, pronounced wrinkles, grooves, and heterogeneous rough regions developed, indicating enhanced stress accumulation and more severe structural reconstruction in the film (Figure 6e,f). It is known that the density of Ga_2_O_3_ (6.44 g/cm^3^ at room temperature) is greater than that of liquid Ga (5.91 g/cm^3^ at room temperature), so the formation of a thicker oxide skin could induce more surface wrinkling or mechanical stress at the liquid-solid interface.

**Figure 7:**
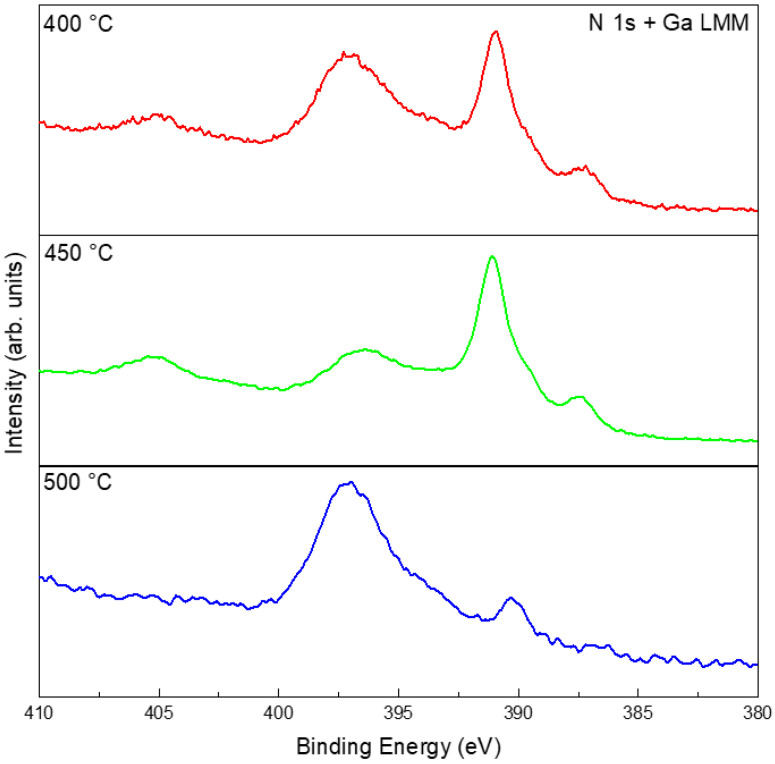
High-resolution XPS spectra of N 1*s* + Ga LMM after treatments in N_2_ at 400, 450, and 500 °C.

The EDS results of the films consistently showed Ga and O as the dominant elements, with strong spatial overlap between the two, whereas no distinct nitrogen signal was observed (Figures S4–S6). Despite the detection of Ga and O with EDS, the Ga_2_O_3_ phase was not present in the XRD patterns (Figure 5) because its quantity was below the instrument’s detection limit. Note that nitrogen was inherently challenging to quantify or map via EDS because of its nature as a very light element. Its low K*α* X-ray energy (0.392 eV) resulted in significant attenuation by the sample matrix and the detection window, often compromising signal accuracy [37]. XPS provided more definitive information on the chemical states: for samples treated in N_2_ at 400, 450, and 500 °C, the high-resolution Ga 3*d* and Ga 2*p* spectra showed peaks mainly at positions corresponding to gallium oxide and elemental Ga, with negligible signals for GaN (Figures S7–S9). Given that the N 1*s* energy window overlaps with that of Ga LMM, it is necessary to compare with the XPS results of the pristine liquid Ga (Figure 3e). The results indicated that the peak at ~397 eV highly likely arose from an oxidation-induced change in the Auger peak profile (Figure 7) [38]. Thus, although the liquid-gallium interface underwent obvious thermal reconstruction in N_2_, the chemical evolution was governed mainly by oxidation rather than nitridation.

Altogether, these characterization results above suggested that, under the present experimental conditions, thermal activation of N_2_ alone was insufficient to induce effective N_2_ fixation and Ga–N bond formation on liquid Ga at 400–500 °C. The surface reaction was instead dominated by oxidation due to the high kinetic barrier for nitridation with N_2_ [12].

To further examine whether stronger thermal activation could promote the reaction between liquid Ga and N_2_, an additional comparison experiment was performed at 800 °C for 6 h (Figures S43–S45). In contrast to the 400–500 °C samples, the 800 °C product exhibited several *h*-GaN-related reflections in XRD, together with detectable Ga–N-related contributions in the Ga 3*d* and Ga 2*p* XPS spectra. Nitrogen was also observed in SEM/EDS mapping. These results indicate that N_2_-derived nitridation of liquid Ga occurred at substantially higher temperatures, whereas it remained kinetically limited in the 400–500 °C range investigated as the focus of this study. Meanwhile, oxide-related signals were still detected, confirming that oxidation remains a competing pathway even at elevated temperature.

### 3.4. Reaction Behavior Under NH_3_

#### 3.4.1. Temperature-Dependent Phase Evolution

**Figure 8:**
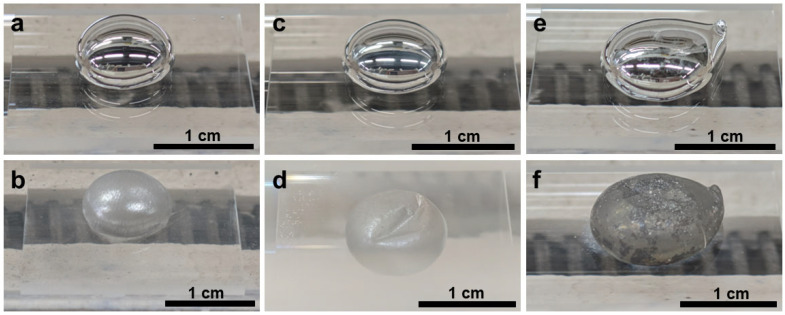
Digital photographs taken at room temperature of liquid Ga samples on quartz glass plates before reactions at (**a**) 400 °C, (**c**) 450 °C, and (**e**) 500 °C and after reactions at (**b**) 400 °C, (**d**) 450 °C, and (**f**) 500 °C in 5%NH_3_–95%Ar.

Compared with N_2_, NH_3_ produced much stronger interfacial modification of the liquid Ga surface (Figure 8). After treatment in 5%NH_3_–95%Ar, the Ga droplets changed from smooth, mirror-like spheres to matte gray structures with roughened and increasingly constrained surfaces, indicating the growth of a more substantial surface reaction layer. The shell-like constraint became more pronounced with increasing temperature.

**Figure 9:**
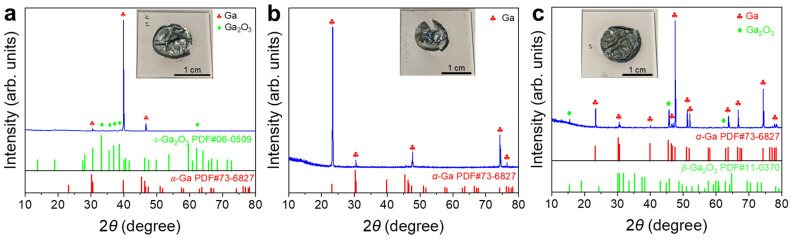
XRD patterns of liquid Ga surface films after heat treatments in 5%NH_3_–95%Ar at (**a**) 400 °C; (**b**) 450 °C; (**c**) 500 °C. Insets are digital photographs of the post-experimental samples with the majority of the liquid Ga underneath withdrawn.

For the sample treated at 400 °C, the XRD pattern was dominated by α-Ga, with peaks at 30.5°, 39.8°, and 46.6° corresponding to the (102), (200), and (022) planes, respectively, while only very weak *ε*-Ga_2_O_3_ signals were detected (Figure 9a). For the sample treated at 450 °C, the XRD pattern remained mainly characteristic of α-Ga (Figure 9b). For the sample treated at 500 °C, the XRD pattern showed the *α*-Ga phase as the dominant phase and *β*-Ga_2_O_3_ as a minor phase (Figure 9c). No crystalline GaN phase was identified in any of three XRD patterns (Figure 9).

For comparison, another experiment of processing liquid Ga in 5%NH_3_–95%Ar at a higher temperature (800 °C) for 6 h was performed. A much thicker and more rigid black surface layer formed, and it was characterized mainly as polycrystalline *h*-GaN, although oxide was still present (Figures S10–S13). The results suggested that the formation of a significant amount of the GaN phase from the reaction of liquid Ga with NH_3_ required a temperature beyond 500 °C.

#### 3.4.2. Surface Morphology and Chemical States

**Figure 10:**
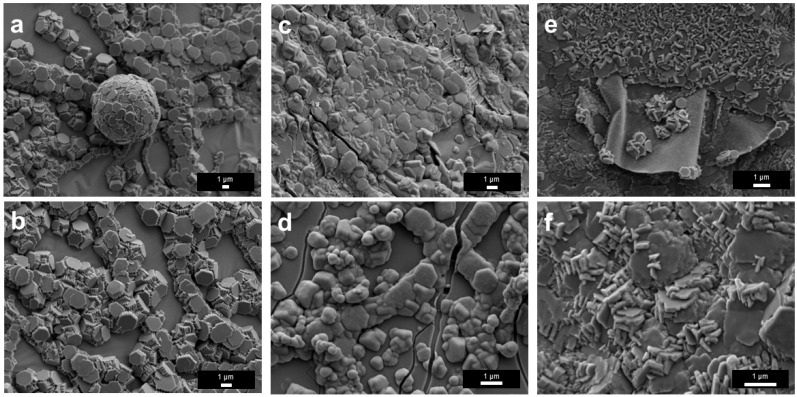
SEM results of liquid Ga films after heat treatments in 5%NH_3_–95%Ar at different temperatures. (**a**,**b**) 400 °C; (**c**,**d**) 450 °C; (**e**,**f**) 500 °C.

The morphology of the surface films treated in 5%NH_3_–95%Ar underwent pronounced evolution with increasing temperature (Figure 10). At 400 °C, the surface was mainly composed of irregular particles and hexagonal plate-like grains (Figure 10a,b). Among these features, the hexagonal plate-like structures are primarily associated with the intrinsic hexagonal symmetry and anisotropic growth behavior of *ε*-Ga_2_O_3_, whereas the spherical structure in Figure 10a might originate from the retraction and droplet formation of liquid Ga, the subsequent constraint imposed by the newly formed surface oxide skin and the extensive formation of early nuclei [34]. It is worth noting that, although many hexagonal crystals were visible in the field of view (Figure 10a,b), no strong corresponding characteristic peaks for *ε*-Ga_2_O_3_ were observed in the XRD analysis (Figure 9), likely because these hexagonal crystals were too thin and did not form a continuous sheet either.

As the temperature increased to 450 °C, the surface gradually became denser, accompanied by the appearance of nodular and cauliflower-like protrusions as well as shrinkage cracks (Figure 10c,d), suggesting that film growth, particle coalescence, and stress accumulation were all intensified. At 500 °C, short plate-like crystals further grew on the sample (Figure 10e,f). Overall, this morphological evolution reflected a complex progressive transition of the liquid Ga film during the NH_3_ experiments.

**Figure 11:**
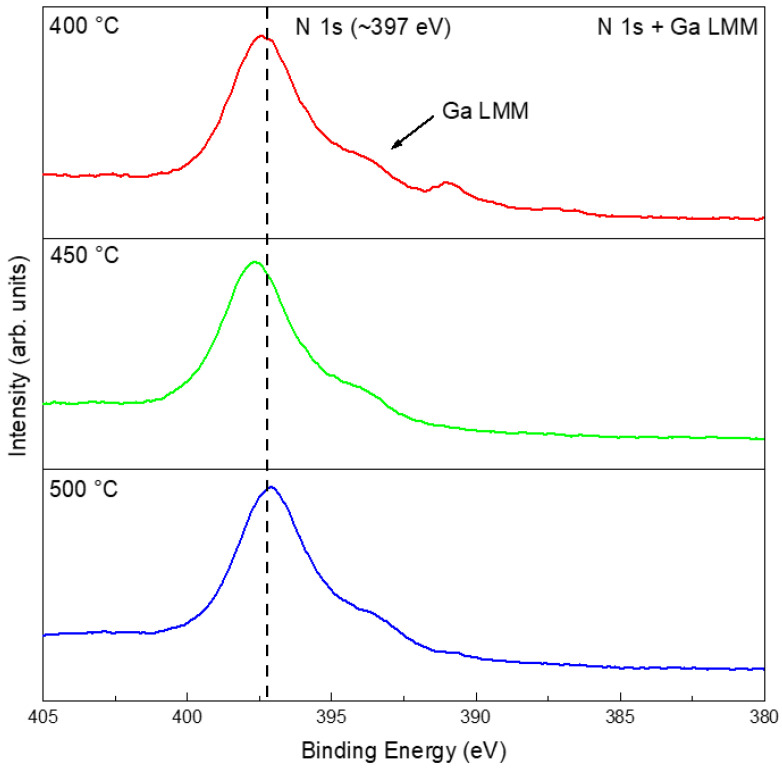
High-resolution XPS results of N 1*s* + Ga LMM after different temperature treatments in 5%NH_3_–95%Ar.

The EDS results show that all NH_3_-treated samples contained Ga, O, and N. Spatially, Ga and O exhibited strong overlap, while N was associated with the surface reaction products, suggesting nitrogen incorporation into the surface layer (Figures S14–S19). At 400–500 °C, the films were still dominated by oxidized gallium species. Compared with the pristine Ga and N_2_-treated samples, however, the NH_3_-treated samples showed additional nitrogen-related and Ga–N-related spectral contributions, as suggested by the combined Ga 3*d*, Ga 2*p*, survey, and N 1*s*/Ga LMM spectra (Figure 11 and Figures S20–S22). Therefore, the liquid Ga surface product in 5%NH_3_–95%Ar is better described as an oxide-dominated interfacial layer containing detectable nitrogen-related species and Ga–N-related contributions, rather than as a fully nitrided or phase-pure GaN product in the 400–500 °C range.

These results suggested that NH_3_ provided a more reactive nitridation environment than N_2_. Nevertheless, the surface oxide and oxygen-related side reactions remain important even in the presence of NH_3_ at the moderate temperatures (400–500 °C). The competition between oxidation and nitridation was therefore a defining feature of the NH_3_-treated system.

#### 3.4.3. Proposed Interfacial Mechanism for Reaction Behavior Under NH_3_

Based on the present ex situ observations and relevant literature, a plausible interfacial pathway for the NH_3_-treated system is proposed as follows. On a clean liquid gallium metal surface, the initial adsorption of NH_3_ may primarily manifest as chemisorption dominated by Lewis acid-base interactions: unsaturated Ga sites act as Lewis acid centers, whose vacant orbitals accept the lone pair electrons from the nitrogen atom of NH_3_, forming a coordinate-type Ga–NH_3_ adsorption complex accompanied by a certain degree of charge transfer and molecular polarization [39,40]. In contrast, van der Waals dispersion and induced dipole forces likely contribute only weakly to the NH_3_ physical adsorption. Subsequently, NH_3_ may undergo progressive dehydrogenation cleavage, forming intermediates such as −NH_2_, −NH, and adsorbed N [41,42]. Simultaneously, released H may recombine on the surface to form H_2_ and desorb. Active surface-adsorbed N may then diffuse into the near-surface region of liquid gallium, forming short-range Ga–N coordination or a nitrogen-enriched dissolution layer. When the local nitrogen activity becomes sufficiently high, GaN nucleation may occur at the gas–liquid interface or at defects or impurities and grow either along the interface or in an island-like manner [43]. The overall reaction rate is likely controlled by the coupling of NH_3_-cracking-derived nitrogen supply and interfacial mass transfer.

**Figure 12:**
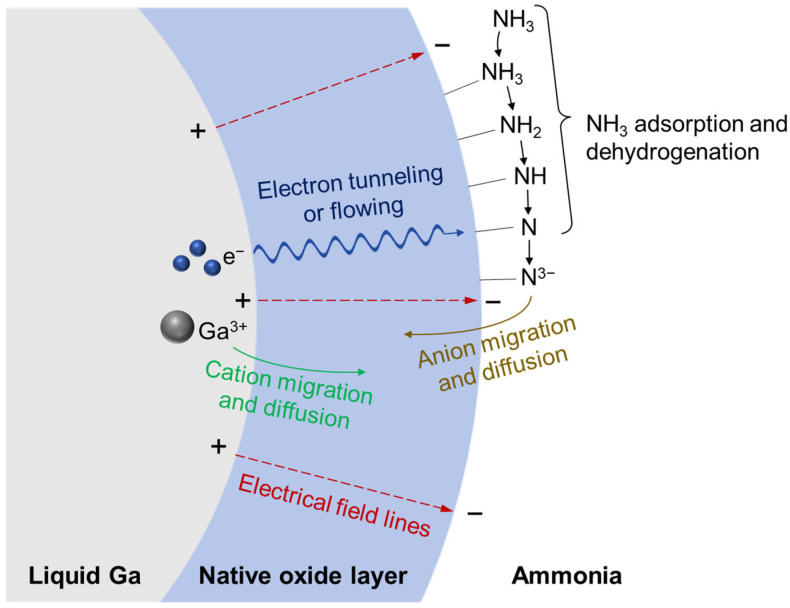
Schematic illustration of the reaction between ammonia and liquid Ga covered with a native oxide layer, analogous to the Cabrera–Mott theory and Wagner’s theory for oxidation.

However, in practical systems, a self-limiting Ga_2_O_3_ oxide layer readily forms on the liquid gallium surface [33,44,45]. Under these conditions, chemisorption dominated by Lewis acid-base interactions likely also occurs first. Ga_2_O_3_, which can also act as a Lewis acid, may accept the lone pair electrons from the NH_3_ nitrogen atom and facilitate NH_3_ chemisorption, followed by progressive dehydrogenation to form N and H adatoms. Subsequent nitridation may proceed analogously to oxidation as described by the Cabrera-Mott theory at relatively low temperatures [46], as illustrated in Figure 12. Adsorbed nitrogen species have a high affinity for electrons. Electrons from liquid Ga may tunnel through the nanometer-thick native oxide layer and combine with adsorbed N outside the oxide, while Ga cations remain inside the oxide. The resulting ionic species may migrate through the oxide layer under the electric field created by the interfacial charge imbalance. At high temperatures, the nitridation behavior may become more similar to the oxidation as described by Wagner’s theory [47]. As the oxide layer gradually grows in thickness, electron tunneling is prohibited but electron transport may become more thermally activated as the oxide becomes more conductive at elevated temperature, and ion transport may increasingly rely on thermal diffusion through the oxide layer (Figure 12). Both the Cabrera-Mott’s field-driven ion migration and the Wagner’s gradient-driven ion diffusion may therefore contribute to nitridation in the NH_3_ system. Alternatively, at high temperatures, oxide-film fracture under stress and liquid-metal flow may locally expose fresh Ga, thereby facilitating local Ga–N bond formation and possible early-stage GaN nucleation to some extent.

### 3.5. Reaction Behavior in the NH_4_Cl: Non-Contact Mode

#### 3.5.1. Morphological Evolution

**Figure 13:**
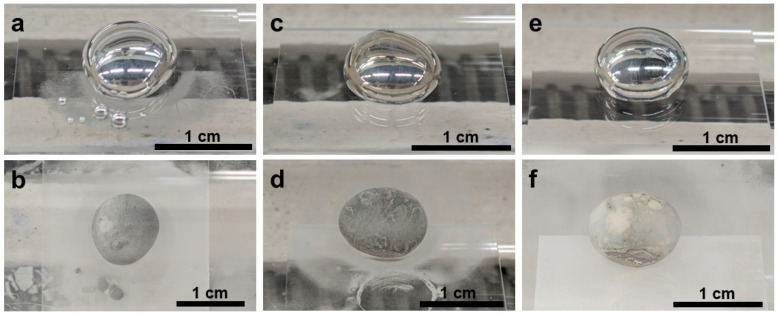
Digital photographs taken at room temperature of liquid Ga samples on quartz glass plates before reactions at (**a**) 400 °C, (**c**) 450 °C, and (**e**) 500 °C and after reactions at (**b**) 400 °C, (**d**) 450 °C, and (**f**) 500 °C using NH_4_Cl in the non-contact mode.

In the non-contact mode, a surface product layer formed on each liquid Ga sample across the whole temperature range of 400–500 °C (Figure 13). As shown in Figure 13d,f, a certain degree of sample displacement was visible. This occurred because the glass substrate was not placed horizontally.

**Figure 14:**
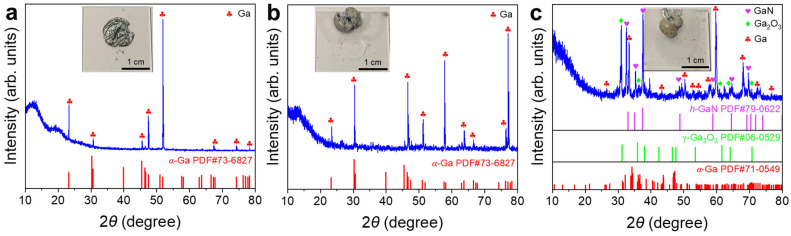
XRD patterns of liquid Ga surface films after thermal treatment using non-contact NH_4_Cl at (**a**) 400 °C, (**b**) 450 °C and (**c**) 500 °C. Insets are digital photographs of the post-experimental samples with the majority of liquid Ga underneath withdrawn.

Figure 14 presents the XRD patterns of the surface films with the liquid Ga underneath withdrawn as a function of the heat treatment temperature under the non-contact NH_4_Cl mode. The results indicated that temperature was a key factor driving the phase transformation. For the samples treated at 400 and 450 °C (Figure 14a,b), the diffraction patterns were mainly dominated by the sharp and intense characteristic peaks of *α*-Ga. For the sample treated at 500 °C (Figure 14c), the XRD pattern of the surface film displayed a highly complex diffractogram. Several reflections could be tentatively assigned to *h*-GaN, suggesting stronger nitridation than at 400–450 °C, although this assignment should be interpreted cautiously. In addition, the α-Ga and *γ*-Ga_2_O_3_ might also be present based on the diffraction peak assignment. The XRD pattern also contained extraneous reflections likely arising from unidentified impurities, highlighting the complexity of the reaction.

#### 3.5.2. Composition and Chemical States

**Figure 15:**
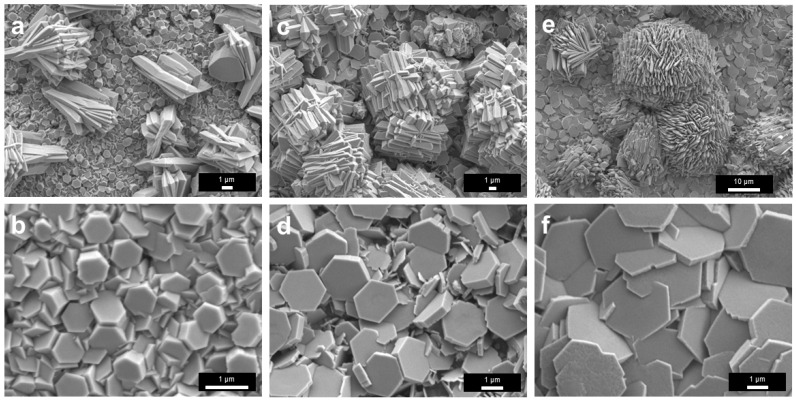
SEM images of liquid Ga surface films after thermal treatment at different temperatures using non-contact NH_4_Cl as the nitrogen source. (**a**,**b**) 400 °C; (**c**,**d**) 450 °C; (**e**,**f**) 500 °C.

With increasing temperature, the surface morphology of the liquid Ga surface films treated in the non-contact NH_4_Cl mode exhibited a pronounced tendency toward coarsening and crystallization. At 400 °C, the surface was mainly covered by densely packed polygonal particles, among which hexagonal plate-like crystals were observable. In addition, micrometer-scale three-dimensional aggregates composed of short rod-like, flake-like, or prismatic microcrystals were also detected (Figure 15a,b). At 450 °C, the hexagonal crystals became larger and more clearly defined, while the three-dimensional aggregates displayed a more developed stacked morphology, indicating enhanced surface diffusion and preferential crystal growth (Figure 15c,d). At 500 °C, some regions were predominantly occupied by relatively large, well-faceted hexagonal plate-like structures, confirming further crystal growth, whereas similar micrometer-scale three-dimensional aggregates became even more pronounced (Figure 15e,f). To further examine the local structure and elemental distribution of the representative microstructures formed at 500 °C, aberration-corrected TEM and STEM-EDS analyses were performed on the non-contact NH_4_Cl sample prepared by FIB milling (Figure S42). The SAED pattern and atomically resolved image indicate local crystallinity with lattice spacings of approximately 0.46 and 0.24 nm, which are consistent with the (002) and (201) planes of *ε*-Ga_2_O_3_, respectively [48]. The corresponding elemental maps show clear Ga and O spatial correspondence, whereas the N signal is weak and lacks clear point-to-point correspondence with the lattice features. These results suggest that at least the analyzed local microstructure was dominated by Ga–O-containing species, consistent with the oxide-rich nature inferred from SEM/EDS and XPS. As shown in Figures S23–S28, EDS consistently identified Ga, O, and N in the film samples obtained from the non-contact experiments. Ga and O showed strong overlap across the observed regions, indicating that the product layer was still mainly composed of Ga–O-containing species. Nitrogen was also detected but usually at lower intensity, suggesting that nitrogen-containing species had participated in the surface reaction (Figures S23–S28).

**Figure 16:**
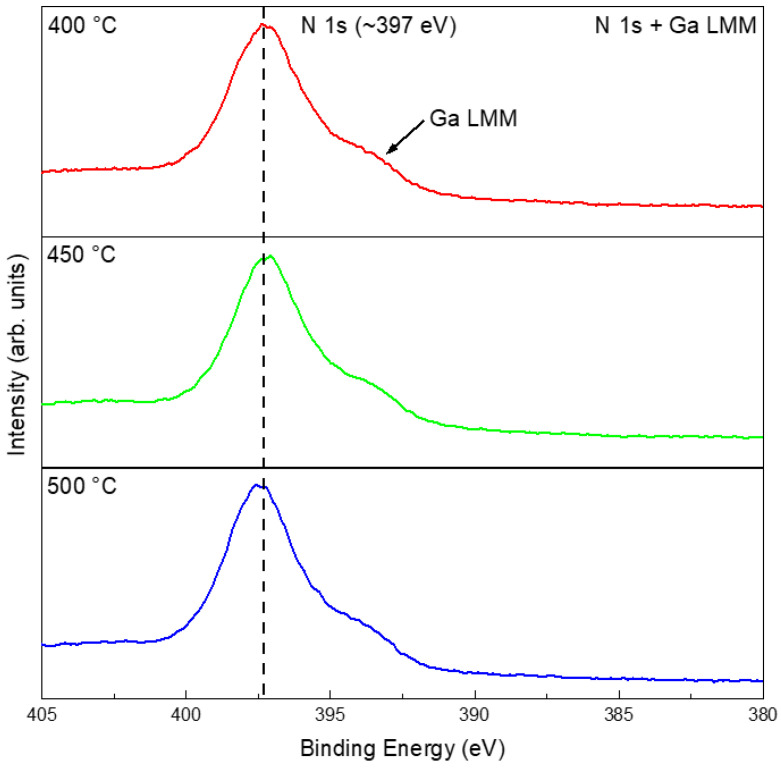
High-resolution XPS spectra of N 1*s* + Ga LMM for the surface films on Ga after heat treatment at 400, 450, and 500 °C using NH_4_Cl as a non-contact nitrogen source.

XPS analysis at 400, 450, and 500 °C indicated oxide-dominated surface chemical states together with nitrogen-related spectral features in the N 1*s*/Ga LMM region (Figure 16 and Figures S29–S31). It should be noted that the feature near ~397 eV cannot be assigned solely to N 1*s* because of its overlap with Ga LMM Auger contributions. Therefore, the interpretation was made by comparing the spectra with pristine Ga and by considering the Ga 3*d*, Ga 2*p*, O 1*s*, and survey spectra together. Survey spectra consistently revealed Ga, O, and N, whereas the Ga 2*p* and Ga 3*d* regions were dominated by oxide-related contributions, with nitride-related components remaining comparatively weak. Thus, in the non-contact NH_4_Cl mode, the product layer is best described as oxide-rich with detectable nitrogen-related species.

#### 3.5.3. Proposed Interfacial Mechanism for Non-Contact NH_4_Cl Reactions

Kovács performed a series of theoretical calculations on the reaction between GaCl_3_ and NH_3_ and revealed that the formation of GaN proceeded through intermediate species [49]. The reaction process between NH_4_Cl and liquid gallium can be summarized by the following chemical equations, where the abbreviations “s”, “g”, and “ads” in parentheses denote the solid, gaseous, and adsorbed states, respectively:

NH_4_Cl(s) ↔ NH_3_(g) + HCl(g) (5)

Ga_2_O_3_(s) + 6HCl(g) → 2GaCl_3_(g/ads) + 3H_2_O(g) (6)

Ga(l) + 3HCl(g) → GaCl_3_(g/ads) + 3/2H_2_(g) (7)

GaCl_3_(g/ads) + NH_3_(g) → Cl_3_GaNH_3_(ads) (8)

Cl_3_GaNH_3_(ads) → GaN(s) + 3HCl(g) (9)

First, NH_4_Cl decomposes to form NH_3_ and HCl (Equation (5)). The generated HCl then reacts with both the oxide layer and the liquid Ga itself to produce GaCl_3_ gas or adsorbate (Equations (6) and (7)). Subsequently, the GaCl_3_ reacts with the NH_3_ released from NH_4_Cl decomposition to form the intermediate gallium trichloride monoammoniate (Cl_3_GaNH_3_) (Equation (8)). Finally, this intermediate undergoes stepwise elimination of HCl, leading to the formation of GaN (Equation (9)). The released HCl can further react with liquid gallium to regenerate GaCl_3_ (Equation (7)), thereby establishing a cyclic process, which continues until the NH_3_ generated from NH_4_Cl decomposition is exhausted and the reaction terminates.

Note that the above process is idealized. In our actual experiments, the amount of NH_3_ and HCl decomposed from the initial 1 g of NH_4_Cl was too small. NH_4_Cl began to decompose at ~338 °C. The NH_4_Cl added could only yield 0.0187 mol (equivalent to 0.419 L at room temperature and 1 atm) of each of NH_3_ and HCl. Under a 20 mL/min Ar flow, the available NH_3_ and HCl reactants were rapidly diluted, resulting in a lack of effective reactants in the later stages of the reaction. Consequently, after the NH_4_Cl was consumed, trace amounts of oxygen in the Ar atmosphere still reacted with Ga to form Ga_2_O_3_.

### 3.6. Reaction Behavior in the NH_4_Cl: Direct-Contact Mode

#### 3.6.1. Enhanced Reaction Intensity and Structural Reconstruction

**Figure 17:**
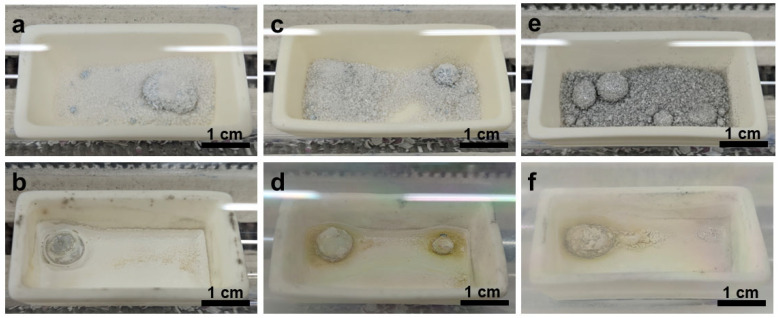
Digital photographs taken at room temperature of liquid Ga samples in the alumina crucible before reaction at (**a**) 400 °C, (**c**) 450 °C, and (**e**) 500 °C and after reaction at (**b**) 400 °C, (**d**) 450 °C, and (**f**) 500 °C using direct-contact NH_4_Cl as the nitrogen source. The glare in some photos originated from the quartz glass casing which was reflective under the lighting.

When NH_4_Cl was brought into direct contact with liquid Ga, the reaction became much more vigorous (Figure 17). Macroscopically, the initially covered droplets transformed into rough gray or pale-yellow solid agglomerates, often accompanied by deposition trails and an obvious change in droplet position or numbers. These morphological features indicated that direct contact markedly accelerated the interfacial reaction, leading to severe disruption of droplet integrity, extensive formation of solid reaction products, and deposition of residues around the droplet.

**Figure 18:**
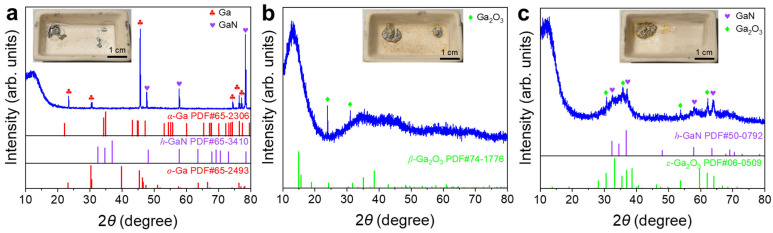
XRD patterns of liquid Ga surface films after thermal treatment using direct-contact NH_4_Cl at (**a**) 400 °C, (**b**) 450 °C and (**c**) 500 °C. Insets are digital photographs of the post-experimental samples with the majority of liquid Ga underneath withdrawn. The broad peaks observed in the 30–45° range in (**b**) and (**c**) correspond to the short-range order structure of liquid Ga.

Figure 18 shows the XRD characteristics of the liquid Ga surface films under direct-contact NH_4_Cl heat treatment. Under the conditions of 400 °C and 500 °C, reflections assignable to *h*-GaN (Figure 18a,c) were observed in the XRD patterns, suggesting that the direct contact between NH_4_Cl and liquid Ga enhanced local nitridation and may have facilitated GaN nucleation and crystallization. However, the system treated at 500 °C was still accompanied by substantial diffraction signals from *ε*-Ga_2_O_3_, as well as a strong background from residual liquid Ga. The coexistence of these multiple phases indicates complex interfacial reactions.

**Figure 19:**
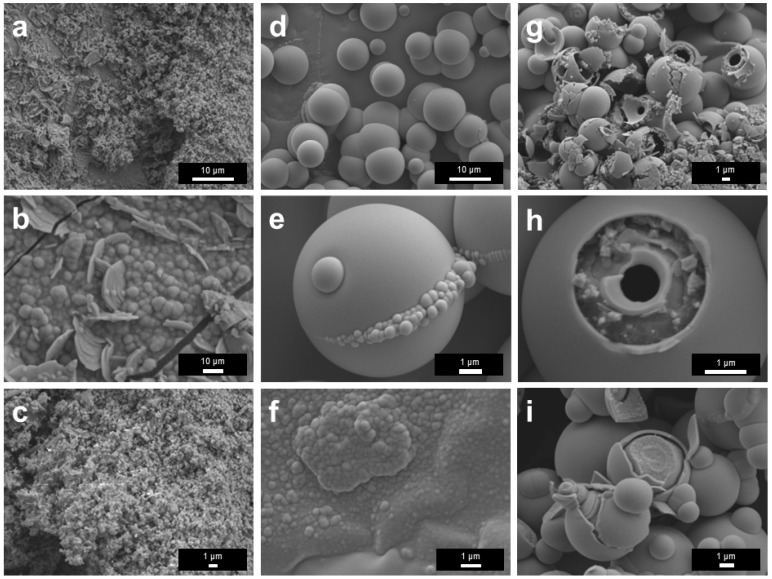
SEM results of Ga films after direct contact with NH_4_Cl and subsequent heat treatment at different temperatures. (**a**–**c**) 400 °C; (**d**–**f**) 450 °C; (**g**–**i**) 500 °C.

Notably, in the samples treated at 450 °C and 500 °C, the signal-to-noise ratios of the XRD patterns were low, and the intensities of the characteristic diffraction peaks were weakened (Figure 18b,c). Combined with the macroscopic photographs of the samples (insets in Figure 18), this behavior might be attributed to the morphological instability of the surface film under highly reactive conditions. As the reaction became more intense, the originally continuous surface layer transformed into spherical substances (Figure 19). Such morphological heterogenization was probably responsible for the diffuse and indistinct signals in the XRD patterns.

#### 3.6.2. Characteristic Morphologies: Spheroids, Shells, and Hollow Structures

The most distinctive feature of the direct-contact-mode samples was the dramatic morphology reconstruction. At 400 °C, the reacted surface mainly consisted of accumulated near-spherical particles mixed with flakes and strips, already indicating strong interfacial reorganization (Figure 19a–c). At 450 °C, spherical particles become the predominant morphology (Figure 19d). Higher-magnification views showed secondary deposition or aggregation around the sphere (Figure 19e). In addition, relatively continuous areas with bumps were also observed (Figure 19f).

At 500 °C, the morphology became even more complex (Figure 19g–i). In addition to large numbers of spherical particles, open hollow spheres, broken shells, and fragmented shell-like residues were clearly observed. Some fractured particles exposed smooth inner surfaces, suggesting that they were hollow rather than solid (Figure 19g,h). In certain regions, open ring-like shells with hierarchical edges were observed, representing advanced shell formation and localized shell failure. These structures implied that the direct-contact reaction at high temperature involved intense interfacial conversion, gas release, possible volatilization/redeposition, shell growth, hollowing, and subsequent mechanical destabilization. For the 500 °C direct-contact NH_4_Cl product, an attempt was also made to prepare cross-sectional TEM specimens using FIB milling. However, the SEM images recorded before and after ion-beam focusing showed a pronounced morphological change in the focused region (Figure S46). The focusing step was performed at an ion-beam accelerating voltage of 30 kV and a high-current setting of 30,000 pA (30 nA). This result indicates that the loosely aggregated or shell-like products formed in the direct-contact sample were sensitive to high-current ion-beam irradiation during FIB operation. Together with the strong residual liquid-Ga background in the XRD pattern and the Ga-rich composition revealed by EDS, this beam sensitivity suggests that at least some spherical or shell-like products may primarily contain liquid Ga, rather than being fully solid oxide or nitride particles. Therefore, unlike the FIB-prepared non-contact NH_4_Cl specimen used for Figure S42, FIB-based preparation of representative cross-sectional TEM specimens from the direct-contact sample was considered unreliable under the present operating conditions. The morphology discussion for the direct-contact sample was therefore mainly based on SEM/EDS, XRD, and XPS characterization.

**Figure 20:**
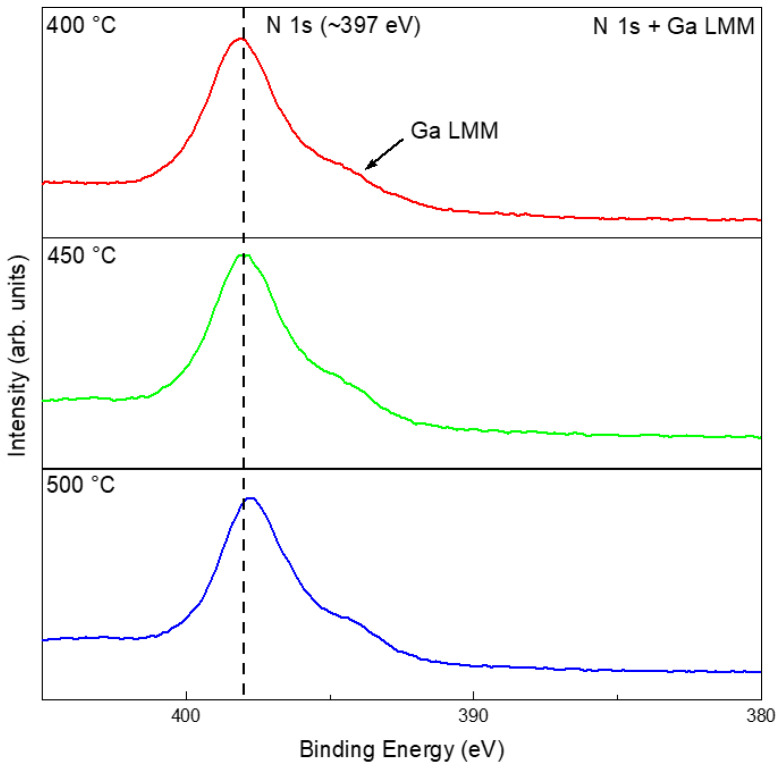
High-resolution XPS spectra of N 1*s* + Ga LMM for the surface films obtained after heat treatment of the mixtures of NH_4_Cl and liquid Ga at 400, 450, and 500 °C.

EDS showed that these direct-contact products were still dominated by Ga and O, with N being clearly detectable but weaker in intensity (Figures S32–S38). XPS further indicated that all direct-contact-mode samples in the 400–500 °C range shared a similar chemical-state signature (Figure 20): oxide-dominated gallium species, detectable nitrogen-related components, and a minor amount of residual metallic Ga (Figures S39–S41). Because of the overlap between N 1*s* and Ga LMM, these nitrogen-related features were interpreted together with the Ga 3*d*, Ga 2*p*, O 1*s*, and survey spectra rather than from the N 1*s*/Ga LMM region alone. Thus, direct contact intensified morphology reconstruction much more strongly than it changed the fundamental chemical competition between oxidation and nitrogen incorporation.

#### 3.6.3. Proposed Interfacial Mechanism for Contact NH_4_Cl Reactions

Like in the non-contact mode, the overall reaction in the direct-contact mode may follow a pathway in which NH_4_Cl first decomposes and then reacts with liquid Ga. The key difference, however, is that in the direct-contact mode, there is no buffer between the decomposed NH_4_Cl species and the liquid Ga; NH_4_Cl decomposition is immediately followed by reaction with Ga. As a result, a locally high-concentration atmosphere of NH_3_ and HCl may be established at the contact interface. GaCl_3_ produced by the reaction of HCl with Ga may subsequently react with NH_3_ to form the intermediate Cl_3_GaNH_3_, which may then release HCl to yield GaN. During the reactions, the large amount of gas generated based on Equations (5)–(7) and (9) may cause rupture of the liquid Ga. The fresh Ga surface in the ruptured regions could serve as favorable sites for local Ga–N bond formation and possible GaN nucleation and growth. In addition, some NH_4_Cl might be trapped inside liquid Ga during rupture; the subsequent NH_4_Cl decomposition could release HCl to corrode the surrounding liquid Ga, and the resulting GaCl_3_ gas might escape with H_2_ or react with the NH_3_, potentially causing the formation of hollow shells or core–shell structures. As the remaining HCl was diluted by Ar, the reactions gradually diminished. After the furnace cooled down, the surface morphology became fixed.

### 3.7. Comparative Discussion: Roles of Nitrogen-Source Reactivity, Oxide Layer, and Contact Mode

Although the reactions were quite complicated, the comparative results across all systems revealed a consistent framework for understanding liquid Ga nitridation behavior.

First, nitrogen-source reactivity was the most direct factor controlling the extent of nitrogen incorporation and Ga–N bond formation. Molecular N_2_ was too inert to effectively nitridize liquid Ga under the present thermal conditions, and the reaction proceeded mainly toward oxide-rich surface layers. NH_3_, by contrast, provided a significantly more reactive nitrogen environment and yielded detectable nitrogen-related signatures and Ga–N-related contributions at 400–500 °C. NH_4_Cl could also introduce nitrogen into the liquid Ga, but its chemistry was accompanied by stronger side reactions and more pronounced local heterogeneity depending on the contact mode.

Second, temperature governed both the extent of interfacial reaction and the crystallization behavior of the products. In the NH_3_ and NH_4_Cl systems, increasing temperature promoted mass transport, surface restructuring, and nitrogen incorporation. In the NH_3_ system, this led to a transition from weakly crystallized, oxide-rich surface products at 400–500 °C to much more pronounced nitridation at 800 °C. The selected 800 °C N_2_ comparison experiment further supports the temperature dependence of the N_2_ system: although N_2_ was ineffective for nitridation at 400–500 °C, more evident GaN-related structural and chemical signatures were observed at 800 °C (Figures S43–S45). This comparison suggests that the inertness of N_2_ in the main temperature window is largely kinetic in nature and can be partially overcome by stronger thermal activation. In the NH_4_Cl systems, higher temperature enhanced hierarchical growth and, especially in direct-contact mode, drove shell formation, hollowing, and local structural collapse.

Third, contact mode strongly modulated local reaction intensity in the NH_4_Cl system. The non-contact mode allowed comparatively mild and spatially more continuous product-layer development. By contrast, the direct-contact mode produced highly localized and vigorous reactions, which favored stronger interface rupture and healing cycles, faster mass transfer, and more dramatic morphology evolution. The emergence of spheroids, open pores, hollow shells, and core–shell structures in the direct-contact mode indicated that local reaction fields dominated the product morphology even when the average chemical composition remained oxide-rich.

Finally, the native oxide layer should not be regarded merely as a passive barrier. The present results suggested a dual role. In the early stage, it can impede direct access to metallic gallium and thereby suppress some nitridation pathways. At the same time, because the oxide layer was thin, defect-prone, and thermochemically labile, it may also participate in adsorption, interfacial charge redistribution, diffusion of Ga^3+^ and N^3−^ ions, and dynamic restructuring. The observed coexistence of oxidation and nitridation signatures across most conditions supported the view that the oxide-covered liquid-gallium interface was a dynamic reaction environment in which oxide-regulated transport and local exposure of fresh gallium jointly controlled the ultimate product evolution.

## 4. Conclusions

In this work, a comparative experimental framework was established to investigate Ga–N bond formation during the reactions of liquid Ga with three representative inorganic nitrogen sources, namely N_2_, NH_3_, and NH_4_Cl. The results showed that the pristine liquid Ga surface was covered by an ultrathin native oxide layer, which played an important role in subsequent interfacial reactions. Under N_2_ at 400–500 °C, the reaction remained oxidation-dominated, with no clear evidence of nitridation under purely thermal conditions. In contrast, NH_3_ promoted nitrogen incorporation more effectively than N_2_, and temperature was identified as a key factor governing interfacial evolution, product crystallization, and morphology development; clear crystalline GaN was observed in the higher-temperature (800 °C) NH_3_ comparison experiment. The selected 800 °C N_2_ comparison experiment further suggested that N_2_-derived nitridation becomes observable only under stronger thermal activation. For the NH_4_Cl-assisted system, the reaction pathway depended strongly on the contact mode: the non-contact configuration led to a relatively moderate reaction and a more continuous surface product layer, whereas the direct-contact configuration induced much stronger interfacial reconstruction and more pronounced morphological heterogeneity, including spheroids, open pores, hollow shells, and core–shell structures. Overall, the present study shows that the reaction behavior of liquid Ga was jointly controlled by the nitrogen source, native oxide layer, temperature, and contact mode, and that the native oxide should not be regarded simply as a passive barrier but as an active interfacial component that may participate in adsorption, charge redistribution, ion transport, and surface reconstruction. These findings provide an experimental basis for understanding nitrogen incorporation and possible Ga–N bond formation at liquid Ga interfaces and offer useful insights for future studies on low-temperature GaN formation and liquid-metal-enabled nitrogen fixation and conversion.

## Figures and Tables

**Table 1:** Experimental details for liquid Ga reactions (ambient pressure, total flow rate 20 mL·min^−1^, heating rate 5 °C·min^−1^, dwell time 6 h).

Label	N Source	Feeding Gas During Purging and Reaction	Sample Holder	T (°C)
C1	N_2_ (control)	N_2_	Quartz plate (25 × 25 mm)	400, 450, 500
A1	5% NH_3_/Ar	5% NH_3_/Ar	Quartz plate (25 × 25 mm)	400, 450, 500
S1	NH_4_Cl-derived (non-contact)	Ar	Alumina crucibles with NH4Cl upstream and Quartz plate support Ga near hot-zone center;	400, 450, 500
S2	NH_4_Cl-derived (contact)	Ar	Alumina crucible; NH_4_Cl in direct contact with Ga	400, 450, 500

## Data Availability

The original contributions presented in this study are included in the article/supplementary material. Further inquiries can be directed to the corresponding author.

## Supplementary Materials

The following supporting information can be downloaded at: https://www.mdpi.com/article/10.3390/ma19101955/s1, Note S1: Gas replacement experiment; Figure S1: Schematic diagram of the pre-installed deoxygenation unit; Figure S2: XRD results of the sample platform; Figure S3: Raman characterization results of liquid Ga; Figure S4: EDS results of the surface film on Ga after heat treatment at 400 °C in an N_2_ atmosphere; Figure S5: EDS results of the surface film on Ga after heat treatment at 450 °C in an N_2_ atmosphere; Figure S6: EDS results of the surface film on Ga after heat treatment at 500 °C in an N_2_ atmosphere; Figure S7: XPS results of the surface film on Ga after heat treatment at 400 °C in an N_2_ atmosphere; Figure S8: XPS results of the surface film on Ga after heat treatment at 450 °C in an N_2_ atmosphere; Figure S9: XPS results of the surface film on Ga after heat treatment at 500 °C in an N_2_ atmosphere; Figure S10: Characterization results of the Ga sample after treatment with 5% NH_3_–95%Ar at 800 °C. No deoxygenation was conducted for the reaction gas in this experiment; Figure S11: SEM and EDS results of the surface film on Ga after heat treatment at 800 °C in 5%NH_3_–95%Ar; Figure S12: XPS results of the surface film on Ga after heat treatment at 800 °C in 5%NH_3_–95%Ar; Figure S13: TEM characterization results of the samples; Figure S14: EDS results of the surface film on Ga after heat treatment at 400 °C in 5%NH_3_–95%Ar; Figure S15: EDS results of the surface film on Ga after heat treatment at 400 °C in 5%NH_3_–95%Ar; Figure S16: EDS results of the surface film on Ga after heat treatment at 450 °C in 5%NH_3_–95%Ar; Figure S17: EDS results of the surface film on Ga after heat treatment at 450 °C in 5%NH_3_–95%Ar; Figure S18: EDS results of the surface film on Ga after heat treatment at 500 °C in 5%NH_3_–95%Ar; Figure S19: EDS results of the surface film on Ga after heat treatment at 500 °C in 5%NH_3_–95%Ar; Figure S20: XPS results of the surface film on Ga after heat treatment at 400 °C in 5%NH_3_–95%Ar; Figure S21: XPS results of the surface film on Ga after heat treatment at 450 °C in 5%NH_3_–95%Ar; Figure S22: XPS results of the surface film on Ga after heat treatment at 500 °C in 5%NH_3_–95%Ar; Figure S23: EDS results of the surface film on Ga after heat treatment at 400 °C using non-contact NH_4_Cl as nitrogen source; Figure S24: EDS results of the surface film on Ga after heat treatment at 400 °C using non-contact NH_4_Cl as nitrogen source; Figure S25: EDS results of the surface film on Ga after heat treatment at 450 °C using non-contact NH_4_Cl as nitrogen source; Figure S26: EDS results of the surface film on Ga after heat treatment at 450 °C using non-contact NH_4_Cl as nitrogen source; Figure S27: EDS results of the surface film on Ga after heat treatment at 500 °C using non-contact NH_4_Cl as nitrogen source; Figure S28: EDS results of the surface film on Ga after heat treatment at 500 °C using non-contact NH_4_Cl as nitrogen source; Figure S29: XPS results of the surface film formed on Ga after heat treatment at 400 °C using NH_4_Cl as a non-contact nitrogen source; Figure S30: XPS results of the surface film formed on Ga after heat treatment at 450 °C using NH_4_Cl as a non-contact nitrogen source; Figure S31: XPS results of the surface film formed on Ga after heat treatment at 500 °C using NH_4_Cl as a non-contact nitrogen source; Figure S32: EDS results of the surface film on Ga after heat treatment at 400 °C under direct contact with NH_4_Cl; Figure S33: EDS results of the surface film on Ga after heat treatment at 400 °C under direct contact with NH_4_Cl; Figure S34: EDS results of the surface film on Ga after heat treatment at 450 °C under direct contact with NH_4_Cl; Figure S35: EDS results of the surface film on Ga after heat treatment at 450 °C under direct contact with NH_4_Cl; Figure S36: EDS results of the surface film on Ga after heat treatment at 450 °C under direct contact with NH_4_Cl; Figure S37: EDS results of the surface film on Ga after heat treatment at 500 °C under direct contact with NH_4_Cl; Figure S38: EDS results of the surface film on Ga after heat treatment at 500 °C under direct contact with NH_4_Cl; Figure S39: XPS results of the surface film formed on Ga after direct mixing of NH_4_Cl with liquid Ga and heat treatment at 400 °C; Figure S40: XPS results of the surface film formed on Ga after direct mixing of NH_4_Cl with liquid Ga and heat treatment at 450 °C; Figure S41: XPS results of the surface film formed on Ga after direct mixing of NH_4_Cl with liquid Ga and heat treatment at 500 °C; Figure S42: Aberration-corrected TEM and STEM-EDS results of a representative FIB-prepared microstructure obtained after heat treatment at 500 °C using NH_4_Cl as a non-contact nitrogen source; Figure S43: XRD pattern of the liquid Ga surface film after heat treatment in N_2_ at 800 °C; Figure S44: SEM and EDS results of the surface film on Ga after heat treatment at 800 °C in an N_2_ atmosphere; Figure S45: XPS results of the surface film on Ga after heat treatment at 800 °C in N_2_; Figure S46. SEM images of the 500 °C direct-contact NH_4_Cl sample recorded during FIB sample preparation.
