# Supplementary material for: Experimental Investigation into the Reactions Between Liquid Gallium and Inorganic Nitrogen Precursors (N2, NH3, and NH4Cl) at 400–500 °C
Source: Materials (Basel). 2026 May 9;19(10):1955. doi: 10.3390/ma19101955 (PMC13208902; doi:10.3390/ma19101955)
Supplement: Supplementary file 1 [file materials-19-01955-s001.zip › materials-4274065-supplementary.pdf]

Supplementary Material

Experimental Investigation into the Reactions between Liquid Gallium and Inorganic Nitrogen Precursors (N_2, NH_3, and NH_4Cl) at 400–500 °C

Yuxi Zheng[1] and Xiaofei Guan[1, *]

[1] School of Physical Science and Technology, ShanghaiTech University, Shanghai, 393 Huaxia Middle Road, Shanghai, 201210, China

* Correspondence: xiaofei@bu.edu; guanxf@shanghaitech.edu.cn

Note S1. Gas purging experiment

Gas purging experiments were conducted to determine the time required to effectively replace the air containing 21% O_2 inside the quartz glass tube reactor (inner diameter: 50 mm; length: 600 mm; internal volume: approx. 1.18 L). Ultra-high-purity argon was fed into the reactor at a flow rate of 20 mL min^{-1}. The outlet gas was passed into a gas chromatograph (GC-2014, Shimadzu™, Japan) equipped with a thermal conductivity detector (TCD), and the signal of O_2 was monitored with time. N_2 (flow rate: 10 mL min^{-1}) was used as an external standard to evaluate the O_2 content in the exit gas. After 7.5 hours of purging (equivalent to a total Ar flow of ~9.0 L, ~7.6 times the volume of the quartz glass tube), the gas chromatograph consistently displayed no O_2 signal for several consecutive runs, suggesting that the O_2 content fell below the instrument's detection limit (0.02% or 200 ppm). In other words, the O_2 content decreased by more than 1050 times. Therefore, it was determined that the air within the quartz glass tube had been effectively replaced. Based on these results, a purging duration of 8 hours was adopted for all subsequent experiments. Note that the starting O_2 content in the quartz glass tube reactors in actual experiments was much lower than that in air because the reactor was assembled in Ar-filled glovebox, transferred out of the glovebox after sealing, and then immediately connected to gas lines.

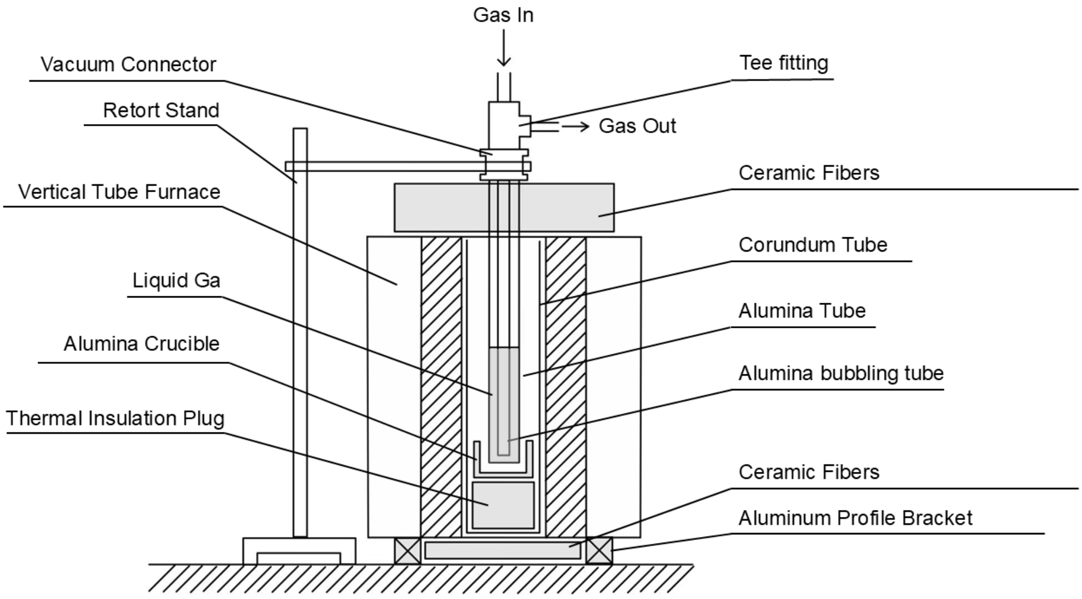

Figure S1. Schematic diagram of the pre-installed deoxygenation unit that was used to reduce the oxygen content in the reaction gas before entering the reactor.

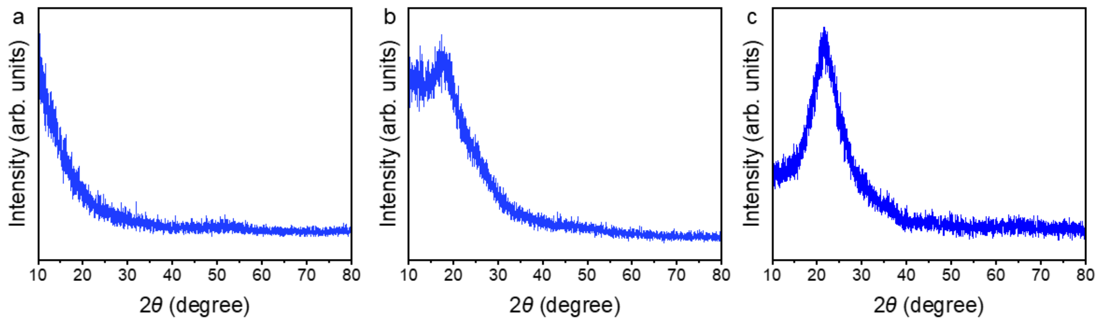

Figure S2. XRD results of the sample holder and quartz glass substrate. (a) Single-crystal silicon sample holder, (b) Silicon sample holder covered with Kapton^{TM} polyimide film. (c) Quartz glass substrate.

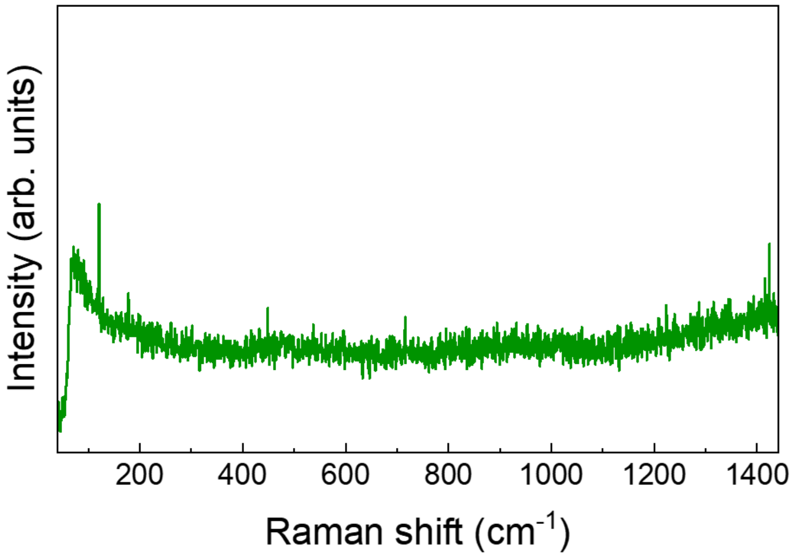

Figure S3. Raman characterization results of the surface of the pristine liquid Ga.

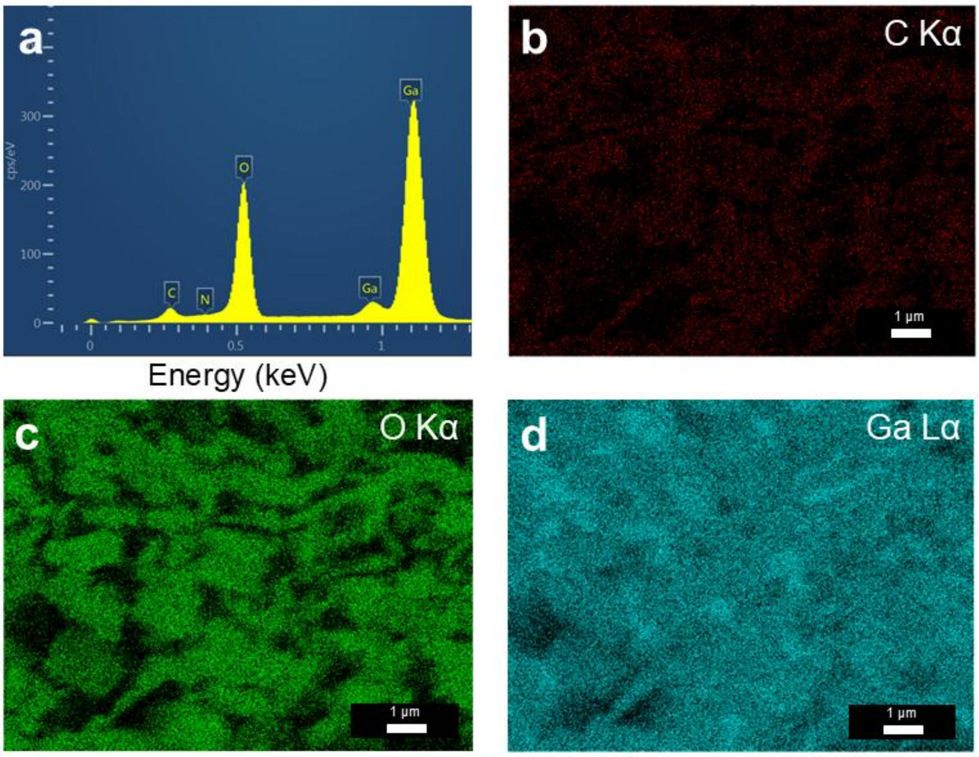

Figure S4. EDS results of the surface film on Ga after heat treatment at 400 °C in an N_2 atmosphere. (a) EDS spectrum acquired from the corresponding area, where Ga and O are the dominant signals with a minor C contribution. (b–d) EDS elemental maps of (b) C, (c) O, and (d) Ga, respectively. O and Ga are broadly and relatively uniformly distributed with a pronounced spatial overlap, indicating that the film is mainly composed of Ga–O species. The weak C signal is likely associated with adventitious carbon contamination introduced during sample preparation or measurement.

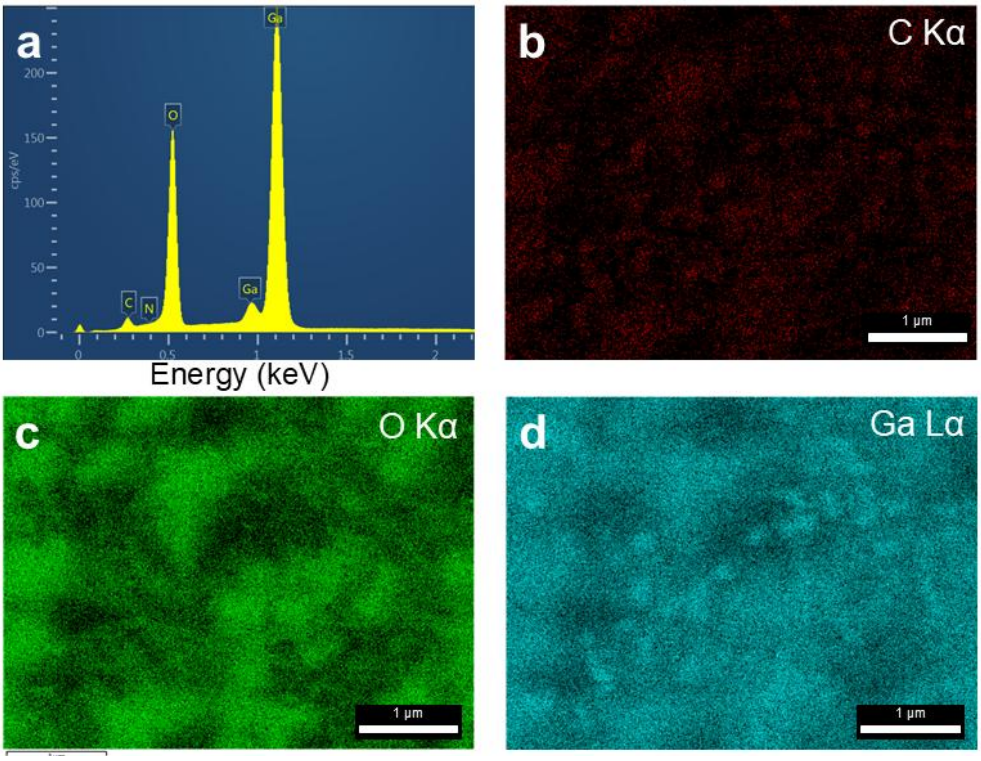

Figure S5. EDS results of the surface film on Ga after heat treatment at 450 °C in an N_2 atmosphere. (a) EDS spectrum acquired from the corresponding area, where Ga and O are the dominant signals with a minor C contribution. (b–d) EDS elemental maps of C (b), O (c), and Ga (d), respectively.

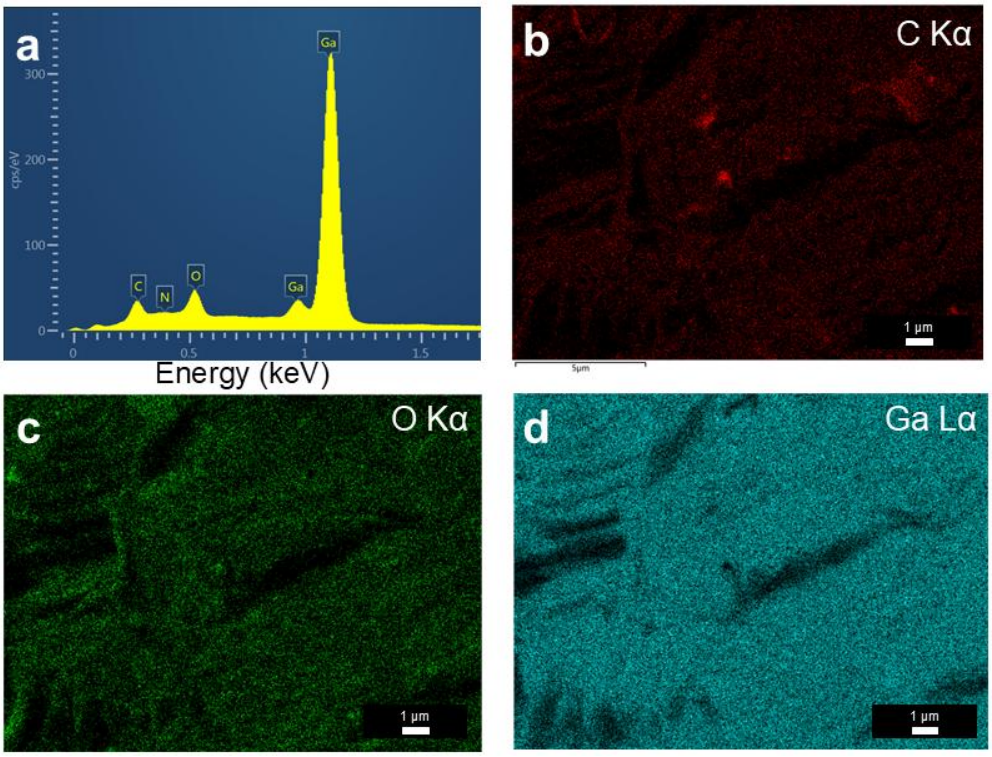

Figure S6. EDS results of the surface film on Ga after heat treatment at 500 °C in an N_2 atmosphere. (a) EDS spectrum acquired from the corresponding area, where Ga is the dominant signal and O is also detected, with a minor C contribution. (b–d) EDS elemental maps of C (b), O (c), and Ga (d), respectively.

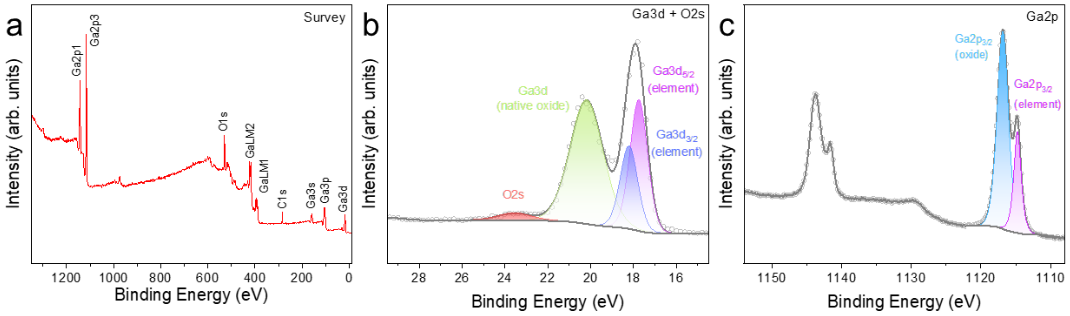

Figure S7. XPS results of the surface film on Ga after heat treatment at 400 °C in an N_2 atmosphere. (a) XPS survey spectrum. (b) High-resolution Ga 3*d* + O 2*s* spectrum with peak fitting. (c) High-resolution Ga 2*p* spectrum with peak fitting.

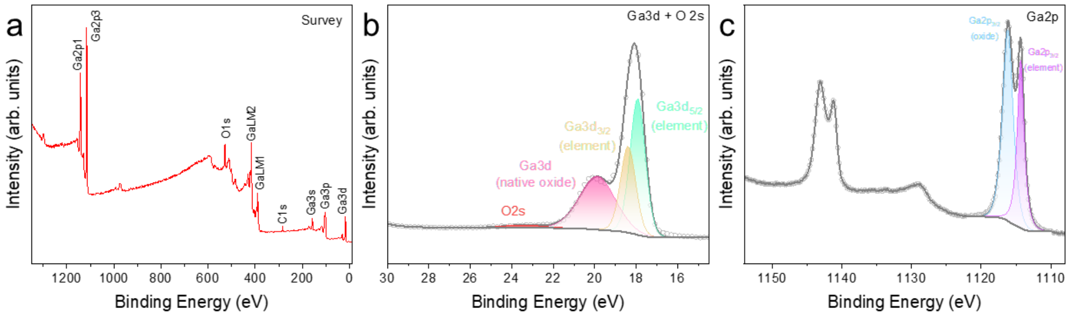

Figure S8. XPS results of the surface film on Ga after heat treatment at 450 °C in an N_2 atmosphere. (a) XPS survey spectrum. (b) High-resolution Ga 3*d* + O 2*s* spectrum with peak fitting. (c) High-resolution Ga 2*p* spectrum with peak fitting.

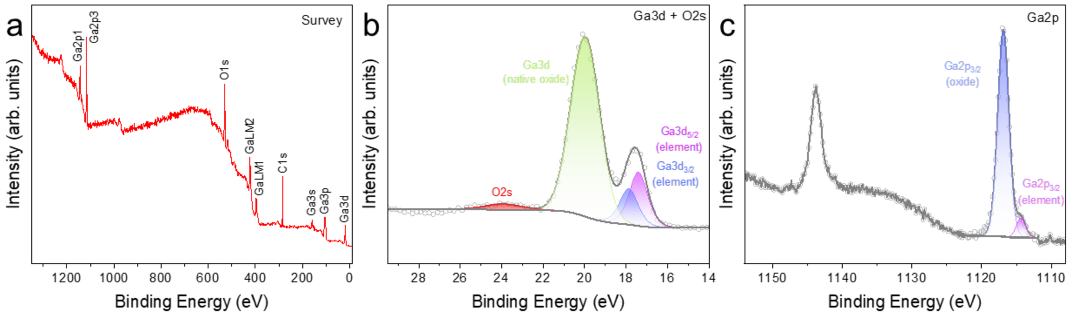

Figure S9. XPS results of the surface film on Ga after heat treatment at 500 °C in an N_2 atmosphere. (a) XPS survey spectrum. (b) High-resolution Ga 3*d* + O 2*s* spectrum with peak fitting. (c) High-resolution Ga 2*p* spectrum with peak fitting.

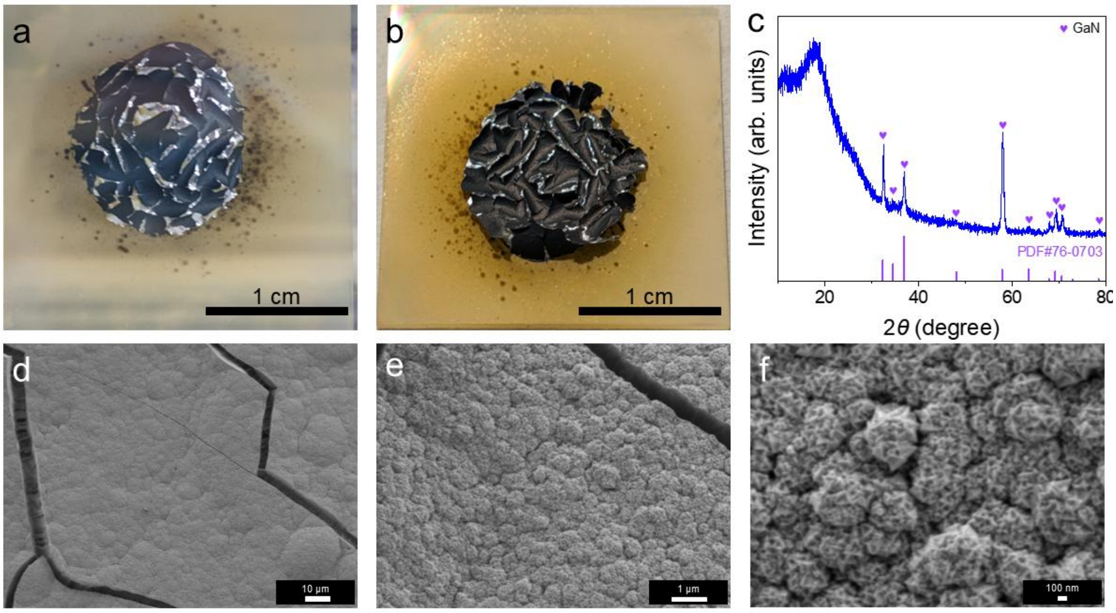

Figure S10. Characterization results of the Ga sample after treatment with 5% NH_3–95%Ar at 800 °C. No deoxygenation was conducted for the reaction gas in this experiment. (a) Digital photograph of the sample after 12 hours of reaction at 800 °C. (b) Digital photograph of the surface film of the same sample after extracting the liquid gallium underneath. The sample was black in the image (b), but when it was still inside a quartz glass tube and photographed from the outside, the sample appeared deep blue in the image (a), which could be caused by light refraction. (c) XRD pattern. The broad peak centered at 18° was attributed to the Kapton^{TM} polyimide film covering the sample during the XRD measurement. (d), (e), (f) SEM images of the surface film.

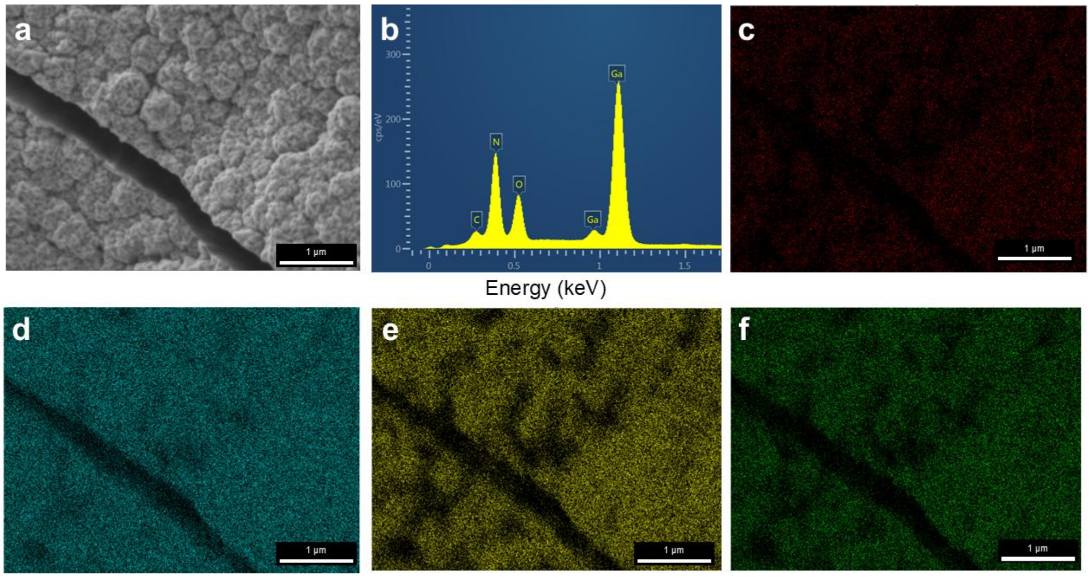

Figure S11. SEM and EDS results of the surface film on Ga after heat treatment at 800 °C in 5%NH_3–95%Ar. (a) SEM image. (b) EDS spectrum acquired from the corresponding area, where Ga and O are the dominant signals with a minor C contribution. (c–f) EDS elemental maps of C (c), Ga (d), N (e), and O (f), respectively.

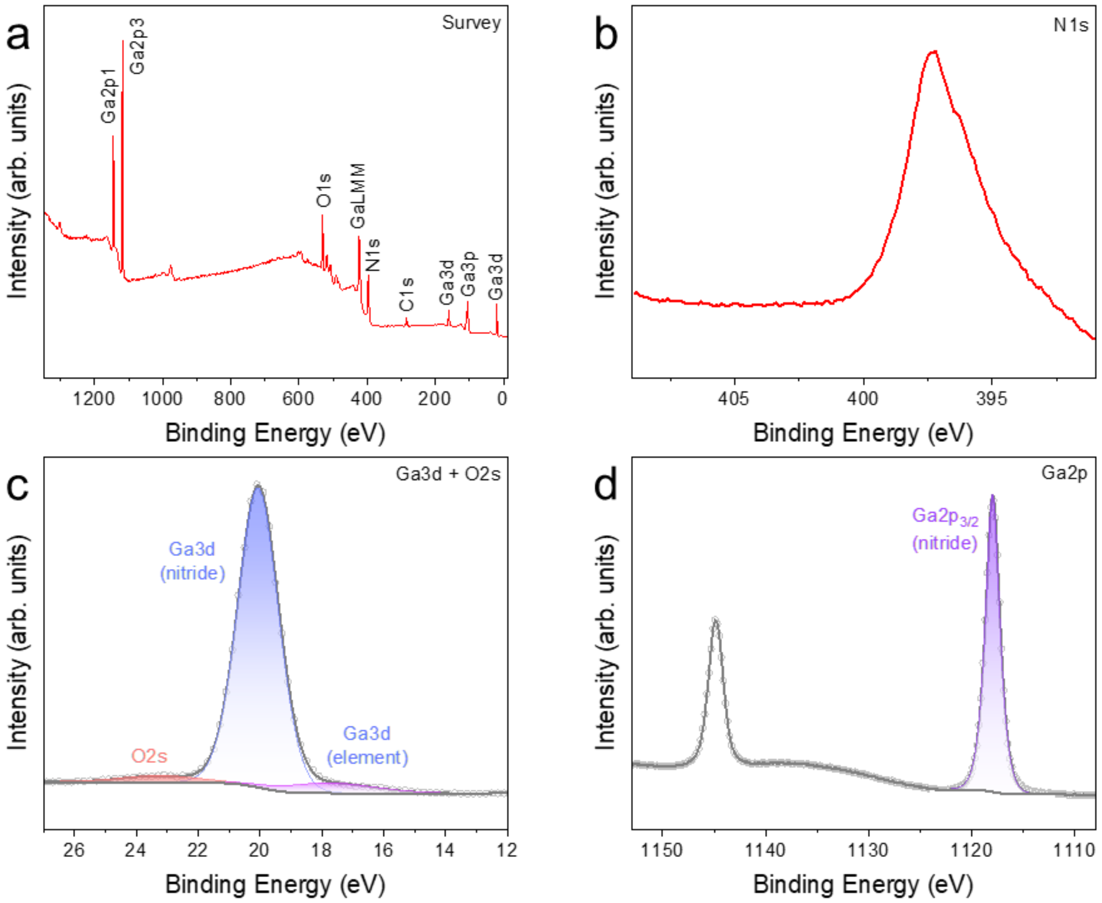

Figure S12. XPS results of the surface film on Ga after heat treatment at 800 °C in 5%NH_3–95%Ar. (a) XPS survey spectrum. (b) High-resolution N 1*s* spectrum. (c) High-resolution Ga 3*d* + O 2*s* spectrum with peak fitting. (d) High-resolution Ga 2*p* spectrum with peak fitting.

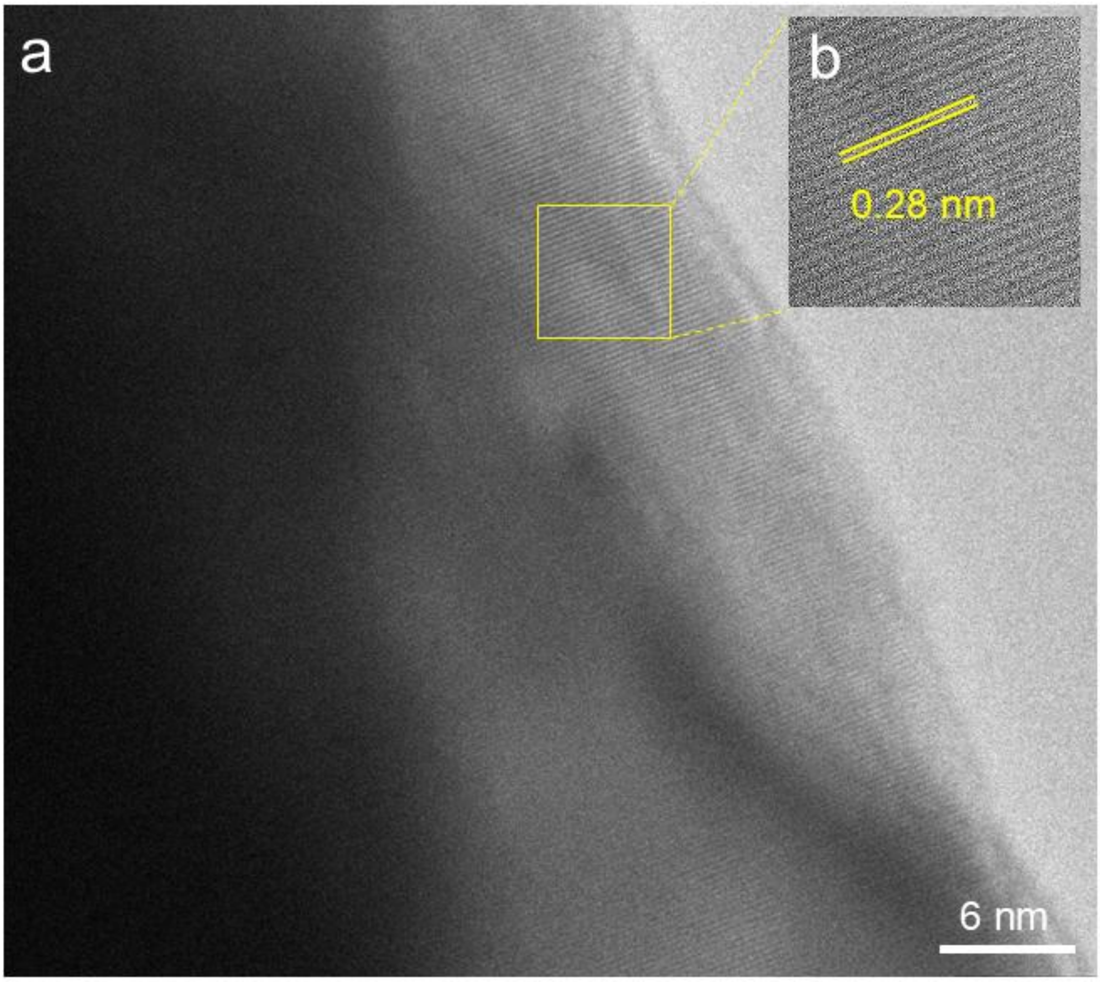

Figure S13. TEM characterization results of the samples: (a) TEM micrograph of the sample treated with 5%NH_3−95%Ar at 800 °C. (b) High-magnification TEM micrograph of the region indicated by the yellow box in Figure (a). The layers stacking with a lattice spacing of ~0.28 nm correspond to the (100) facet of GaN.

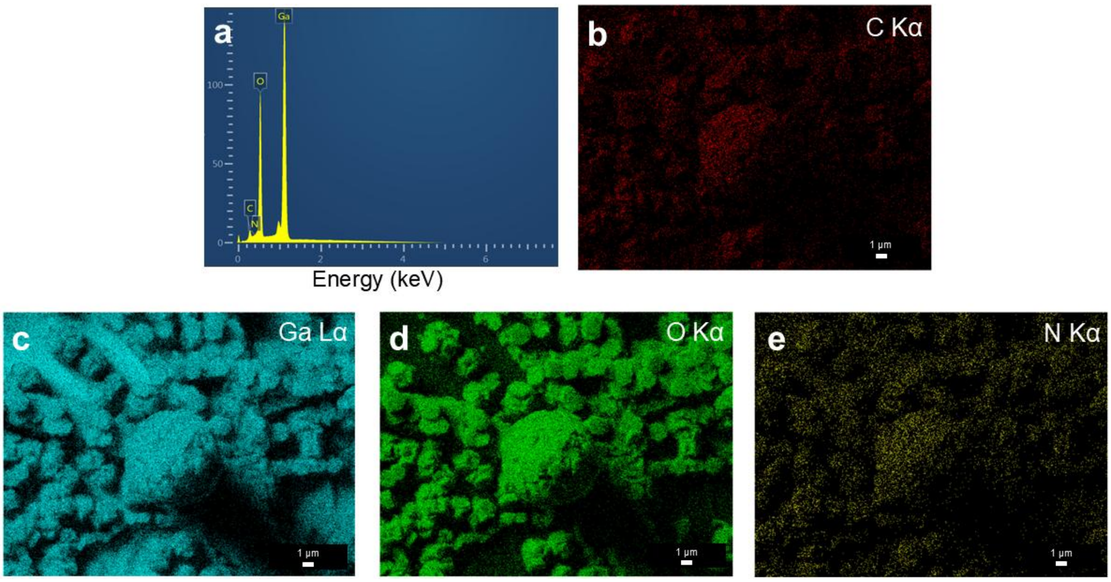

Figure S14. EDS results of the surface film on Ga after heat treatment at 400 °C in 5%NH_3−95%Ar. (a) EDS spectrum of the corresponding region, revealing a dominant Ga signal accompanied by O, N, and minor C signals. (b−e) EDS elemental distribution maps of (b) C, (c) Ga, (d) O, and (e) N, respectively.

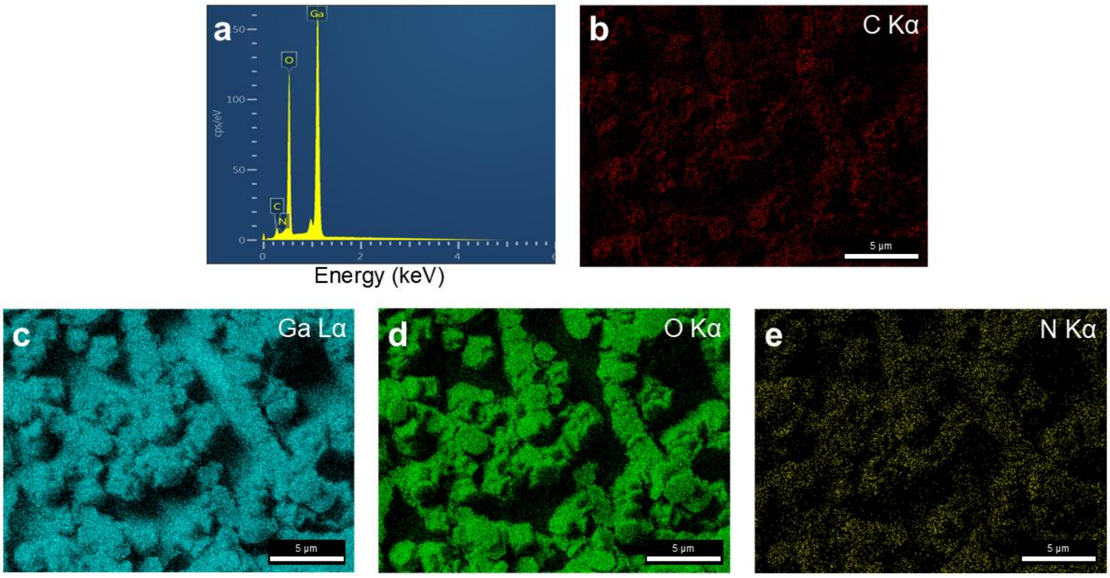

Figure S15. EDS results of the surface film on Ga after heat treatment at 400 °C in 5%NH_3−95%Ar. (a) Corresponding EDS spectrum, showing the sample dominated by Ga signals accompanied by O, N, and trace C signals. (b−e) EDS area maps: elemental distributions of C (b), Ga (c), O (d), and N (e), respectively.

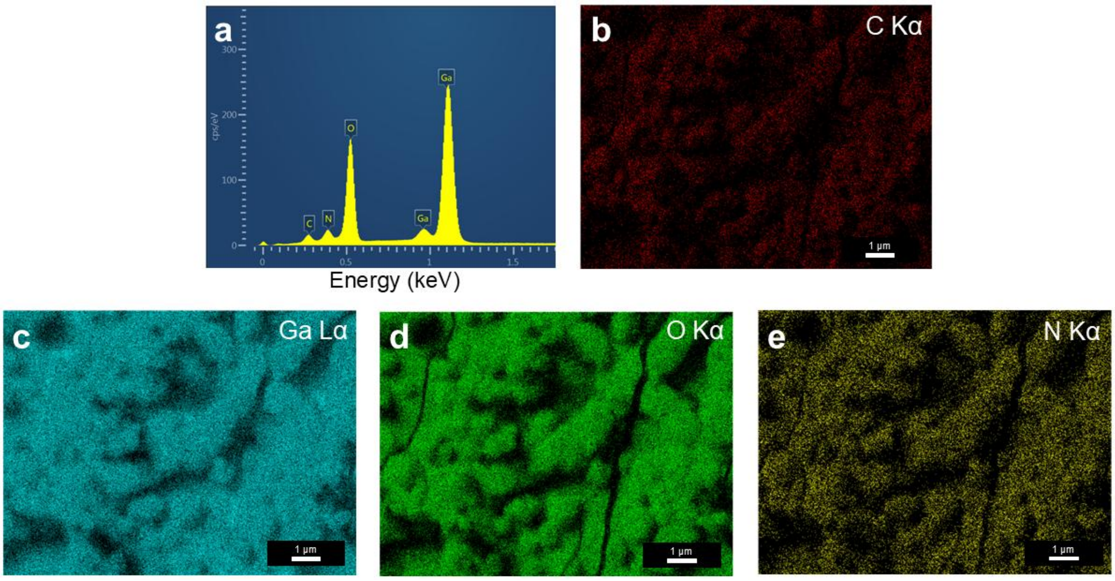

Figure S16. EDS results of the surface film on Ga after heat treatment at 450 °C in 5%NH_3–95%Ar. (a) EDS spectrum of the corresponding region, showing a dominant Ga signal with accompanying O, N, and minor C signals. (b–e) EDS elemental distribution maps of (b) C, (c) Ga, (d) O, and (e) N, respectively.

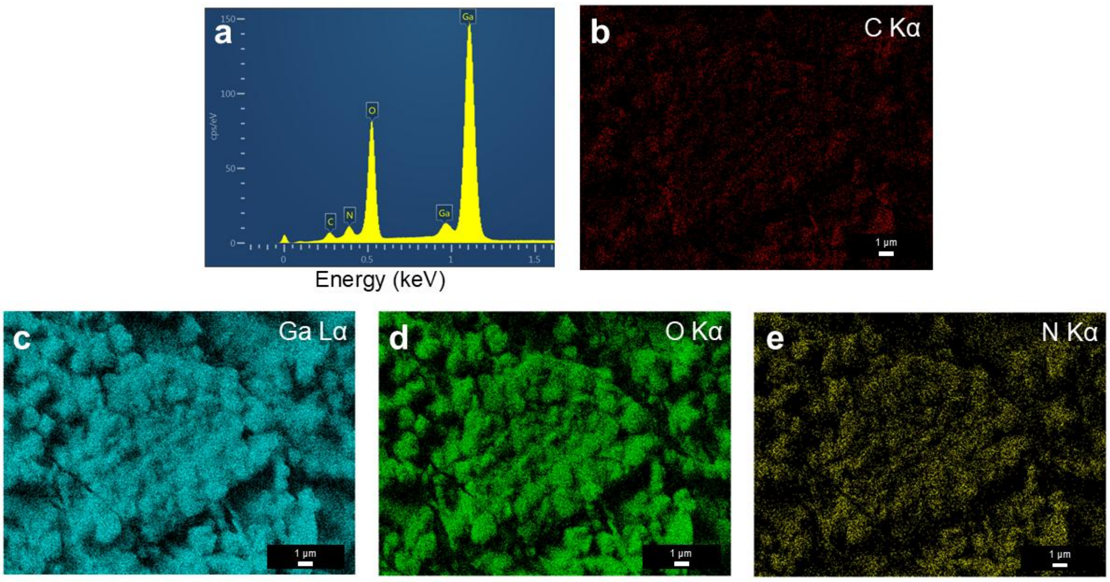

Figure S17. EDS results of the surface film on Ga after heat treatment at 450 °C in 5%NH_3–95%Ar. (a) EDS spectrum of the corresponding region shows the sample dominated by Ga signals, accompanied by O, N, and trace C signals. (b–e) EDS elemental distribution maps of (b) C, (c) Ga, (d) O, and (e) N, respectively.

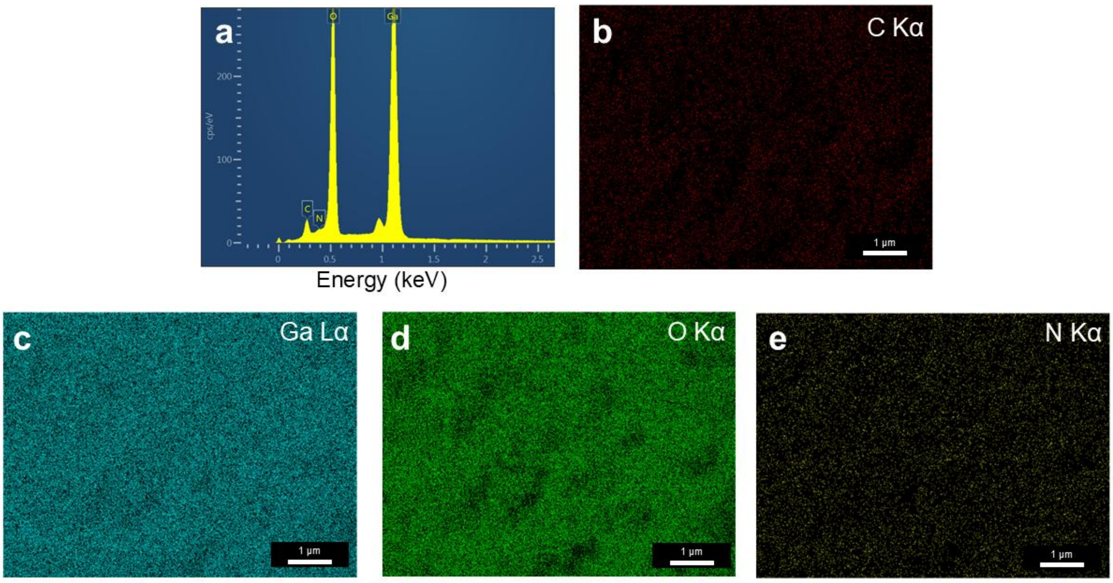

Figure S18. EDS results of the surface film on Ga after heat treatment at 500 °C in 5%NH_3−95%Ar. (a) EDS spectrum of the corresponding region shows the sample dominated by Ga signals, accompanied by O, N, and trace C signals. (b−e) EDS elemental distribution maps of (b) C, (c) Ga, (d) O, and (e) N, respectively.

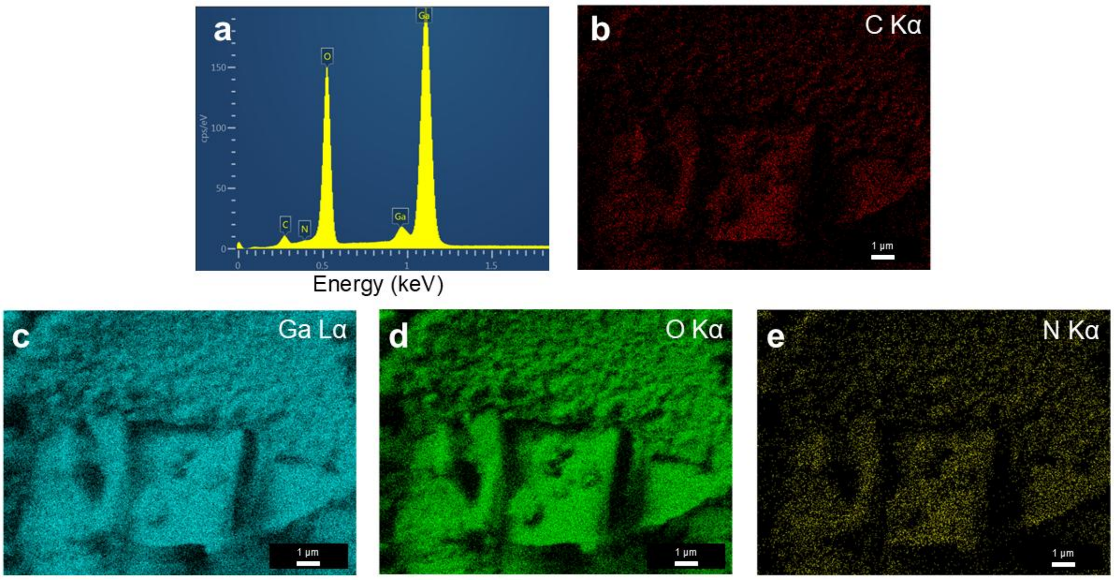

Figure S19. EDS results of the surface film on Ga after heat treatment at 500 °C in 5%NH_3−95%Ar. (a) EDS spectrum of the corresponding region, showing a dominant Ga signal accompanied by O, N, and minor C signals; (b−e) EDS elemental distribution maps of (b) C, (c) Ga, (d) O, and (e) N, respectively.

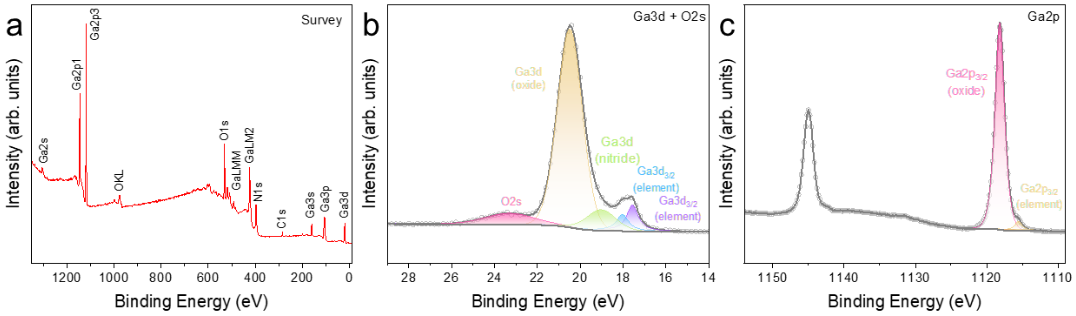

Figure S20. XPS results of the surface film on Ga after heat treatment at 400 °C in 5%NH_3–95%Ar. (a) XPS survey spectrum. (b) High-resolution Ga 3*d* + O 2*s* spectrum with peak fitting. (c) High-resolution Ga 2*p* spectrum with peak fitting.

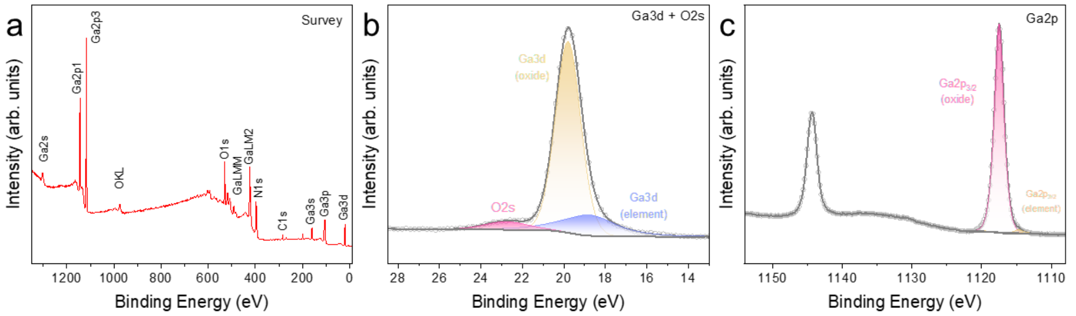

Figure S21. XPS results of the surface film on Ga after heat treatment at 450 °C in 5%NH_3–95%Ar. (a) XPS survey spectrum. (b) High-resolution Ga 3*d* + O 2*s* spectrum with peak fitting. (c) High-resolution Ga 2*p* spectrum with peak fitting.

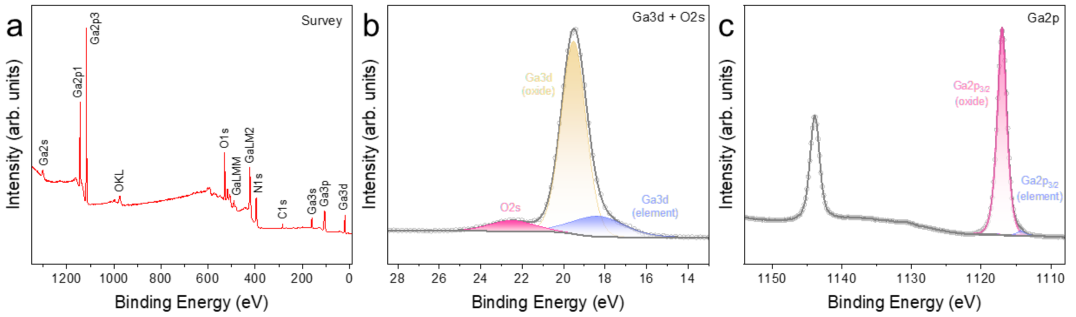

Figure S22. XPS results of the surface film on Ga after heat treatment at 500 °C in 5%NH_3–95%Ar. (a) XPS survey spectrum. (b) High-resolution Ga 3*d* + O 2*s* spectrum with peak fitting. (c) High-resolution Ga 2*p* spectrum with peak fitting.

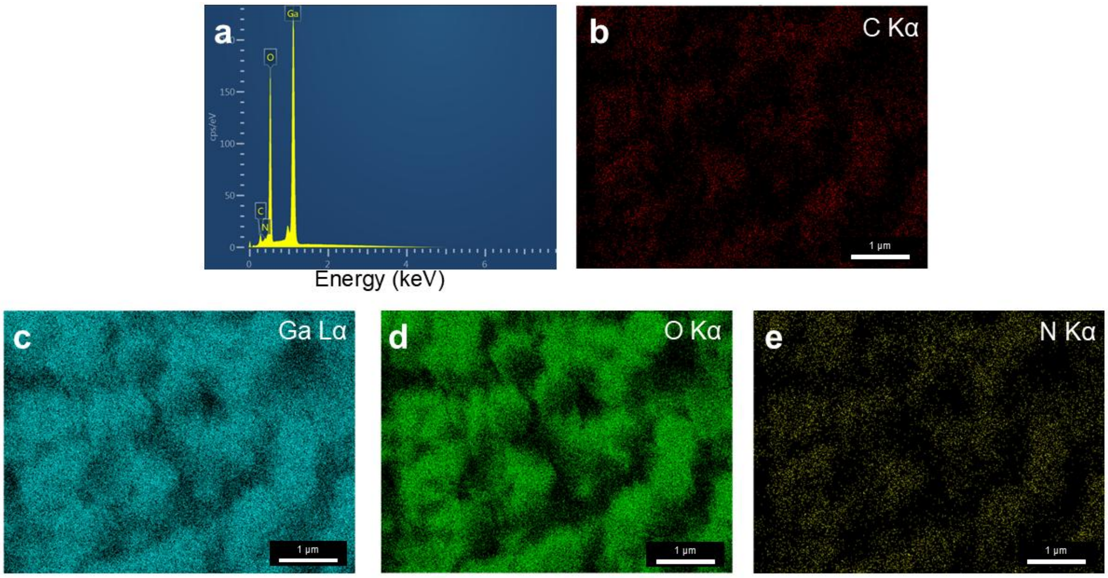

Figure S23. EDS results of the surface film on Ga after heat treatment at 400 °C using non-contact NH_4Cl as nitrogen source. (a) Corresponding EDS spectrum, showing the sample dominated by Ga signals alongside O, N, and minor C signals. (b–e) EDS elemental distribution maps of (b) C, (c) Ga, (d) O, and (e) N.

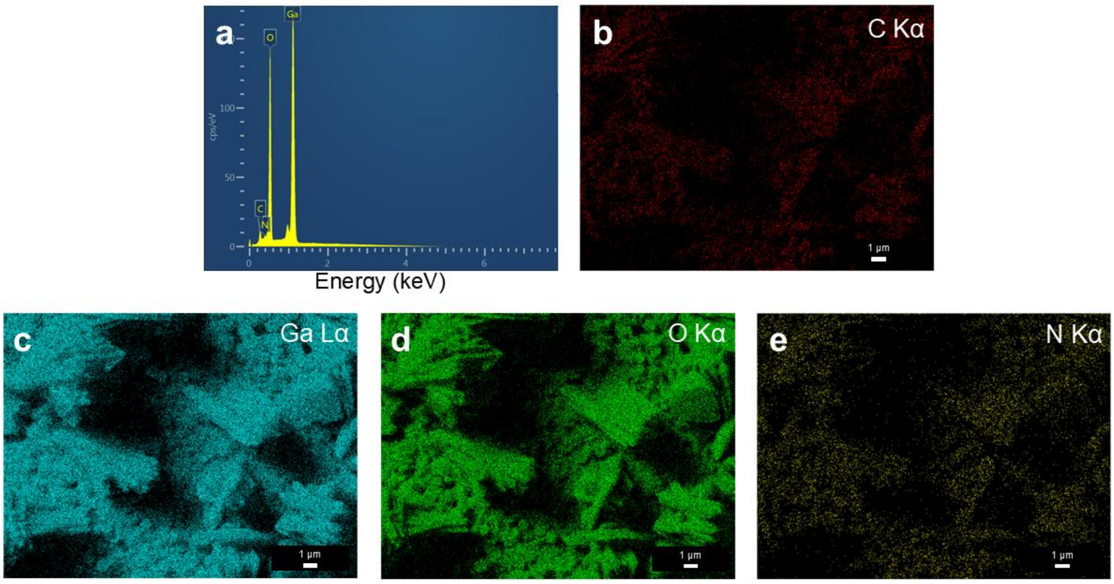

Figure S24. EDS results of the surface film on Ga after heat treatment at 400 °C using non-contact NH_4Cl as nitrogen source. (a) Corresponding EDS spectrum, showing the sample dominated by Ga signals alongside O, N, and minor C signals. (b–e) EDS elemental distribution maps of (b) C, (c) Ga, (d) O, and (e) N.

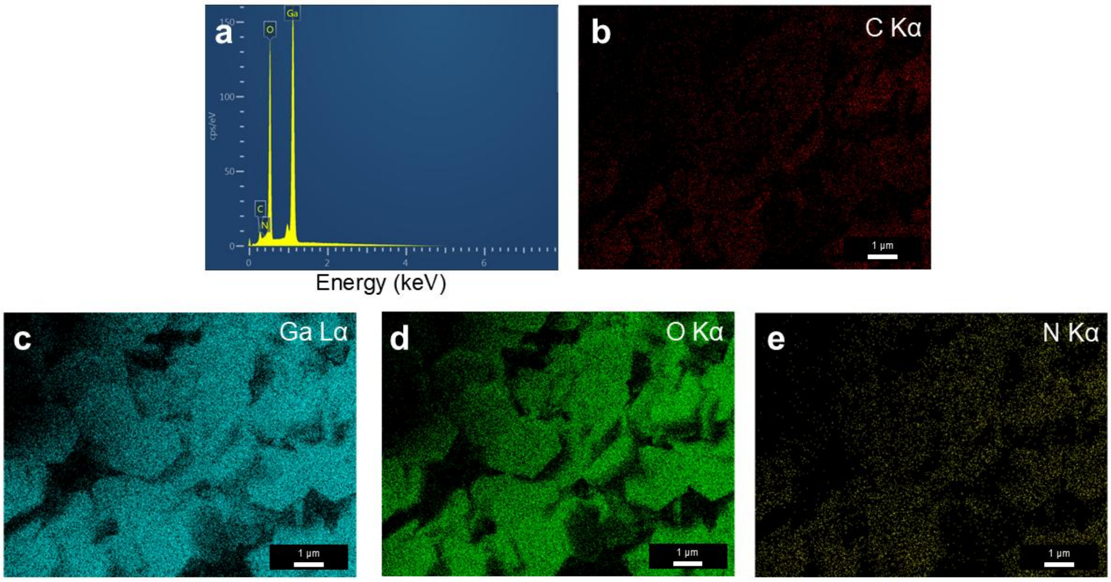

Figure S25. EDS results of the surface film on Ga after heat treatment at 450 °C using non-contact NH_4Cl as nitrogen source. (a) Corresponding EDS spectrum, showing the sample dominated by Ga signals alongside O, N, and minor C signals. (b–e) EDS elemental distribution maps of (b) C, (c) Ga, (d) O, and (e) N.

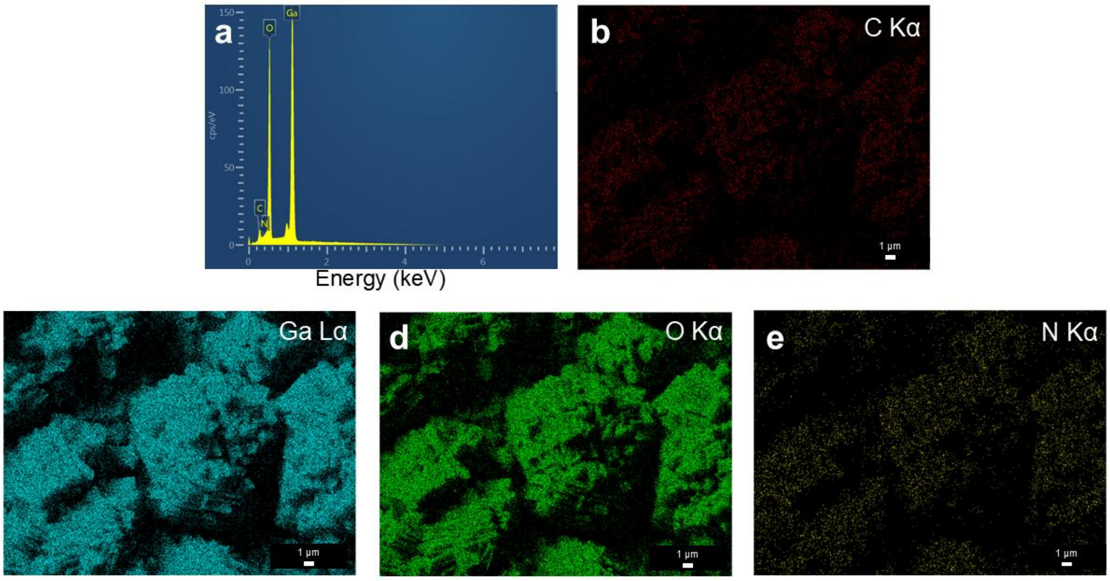

Figure S26. EDS results of the surface film on Ga after heat treatment at 450 °C using non-contact NH_4Cl as nitrogen source. (a) Corresponding EDS spectrum, showing the sample dominated by Ga signals alongside O, N, and minor C signals. (b–e) EDS elemental distribution maps of (b) C, (c) Ga, (d) O, and (e) N.

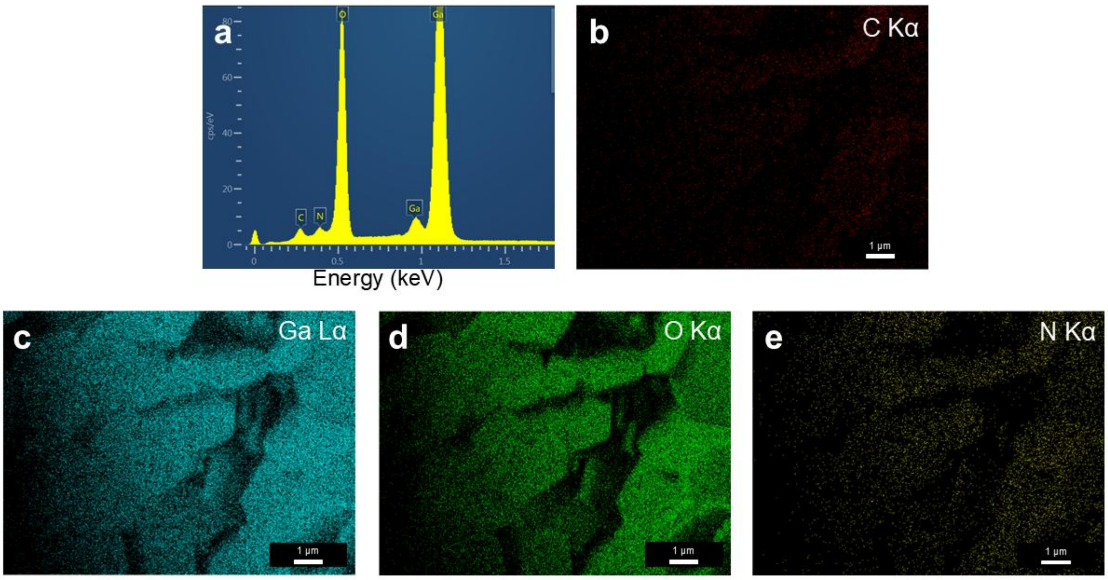

Figure S27. EDS results of the surface film on Ga after heat treatment at 500 °C using non-contact NH_4Cl as nitrogen source. (a) Corresponding EDS spectrum, showing the sample dominated by Ga signals alongside O, N, and minor C signals. (b–e) EDS elemental distribution maps of (b) C, (c) Ga, (d) O, and (e) N.

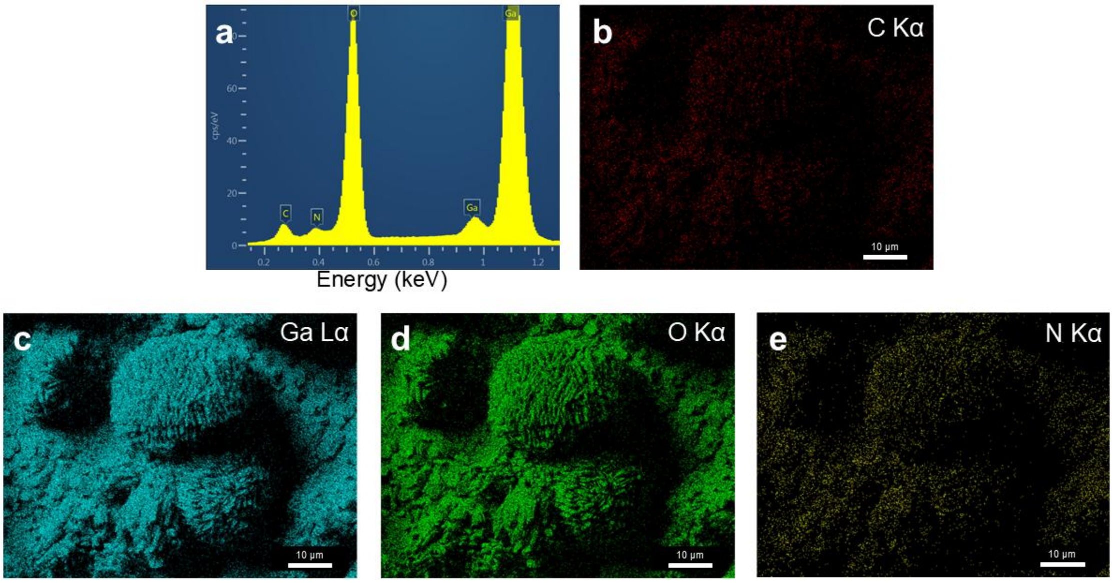

Figure S28. EDS results of the surface film on Ga after heat treatment at 500 °C using non-contact NH_4Cl as nitrogen source. (a) Corresponding EDS spectrum, showing the sample dominated by Ga signals alongside O, N, and minor C signals. (b–e) EDS elemental distribution maps of (b) C, (c) Ga, (d) O, and (e) N.

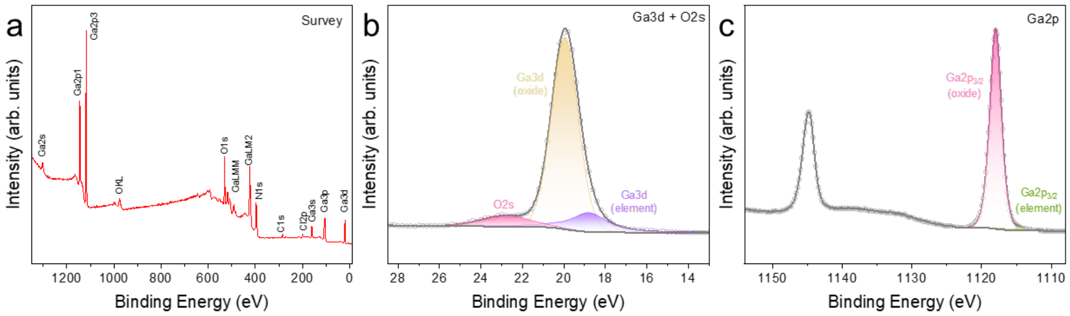

Figure S29. XPS results of the surface film formed on Ga after heat treatment at 400 °C using NH_4Cl as a non-contact nitrogen source. (a) XPS survey spectrum. (b) High-resolution Ga 3*d* + O 2*s* spectrum with peak fitting. (c) High-resolution Ga 2*p* spectrum with peak fitting.

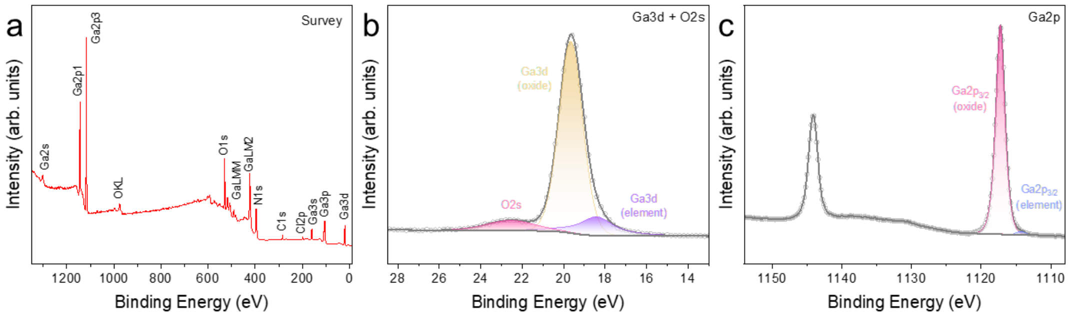

Figure S30. XPS results of the surface film formed on Ga after heat treatment at 450 °C using NH_4Cl as a non-contact nitrogen source. (a) XPS survey spectrum. (b) High-resolution Ga 3*d* + O 2*s* spectrum with peak fitting. (c) High-resolution Ga 2*p* spectrum with peak fitting.

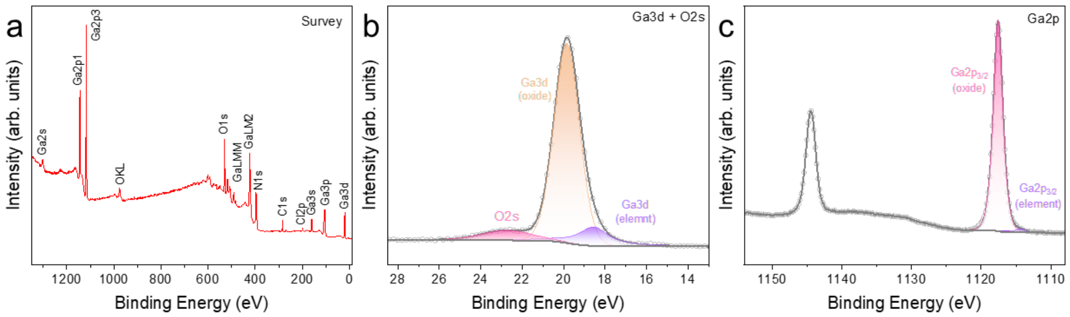

Figure S31. XPS results of the surface film formed on Ga after heat treatment at 500 °C using NH_4Cl as a non-contact nitrogen source. (a) XPS survey spectrum. (b) High-resolution Ga 3*d* + O 2*s* spectrum with peak fitting. (c) High-resolution Ga 2*p* spectrum with peak fitting.

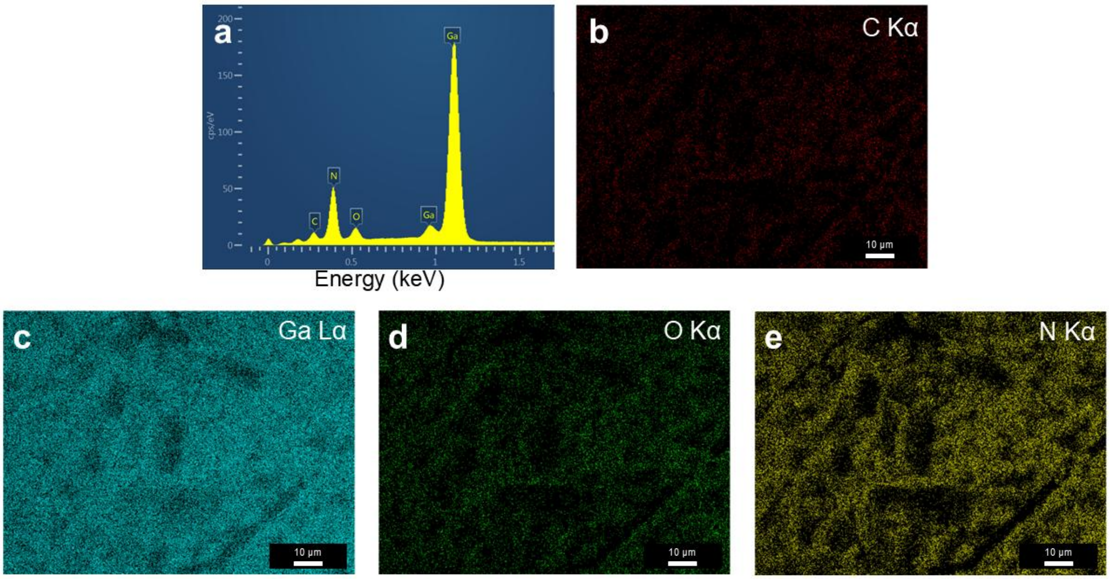

Figure S32. EDS results of the surface film on Ga after heat treatment at 400 °C using direct-contact NH_4Cl as nitrogen source. (a) Corresponding EDS spectrum, showing the sample dominated by Ga signals alongside O, N, and minor C signals. (b−e) EDS elemental distribution maps of (b) C, (c) Ga, (d) O, and (e) N.

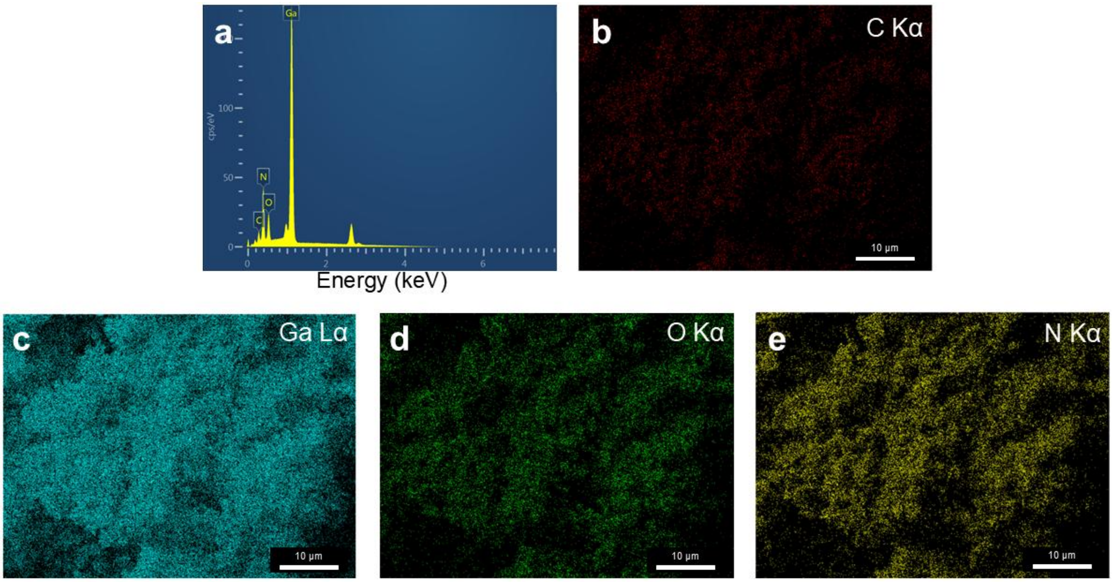

Figure S33. EDS results of the surface film on Ga after heat treatment at 400 °C using direct-contact NH_4Cl as nitrogen source. (a) Corresponding EDS spectrum, showing the sample dominated by Ga signals alongside O, N, and minor C signals. (b−e) EDS elemental distribution maps of (b) C, (c) Ga, (d) O, and (e) N.

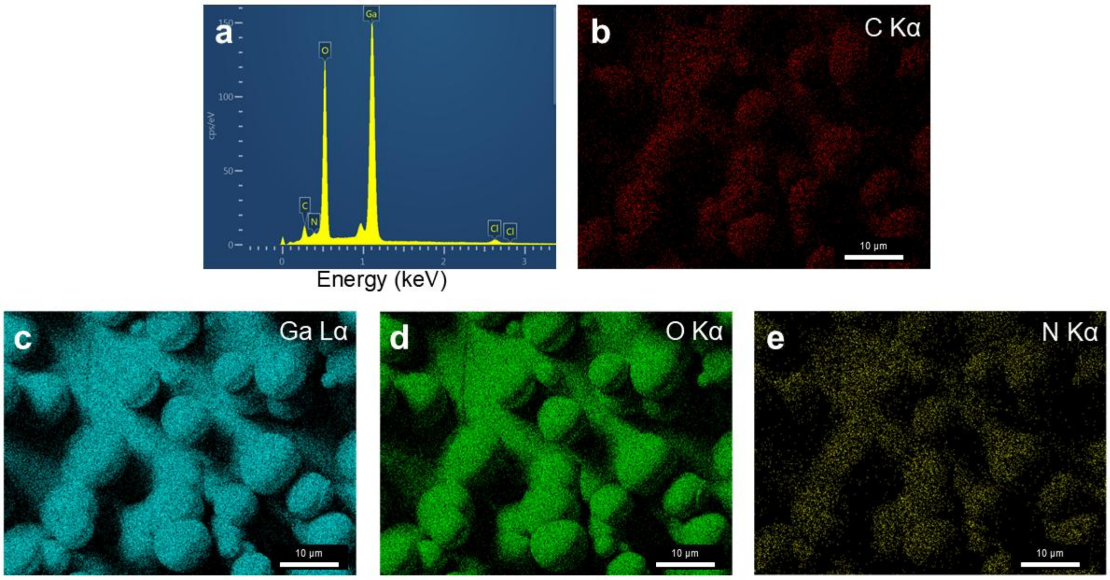

Figure S34. EDS results of the surface film on Ga after heat treatment at 450 °C using direct-contact NH_4Cl as nitrogen source. (a) Corresponding EDS spectrum, showing the sample dominated by Ga signals alongside O, N, and minor C signals. (b−e) EDS elemental distribution maps of (b) C, (c) Ga, (d) O, and (e) N.

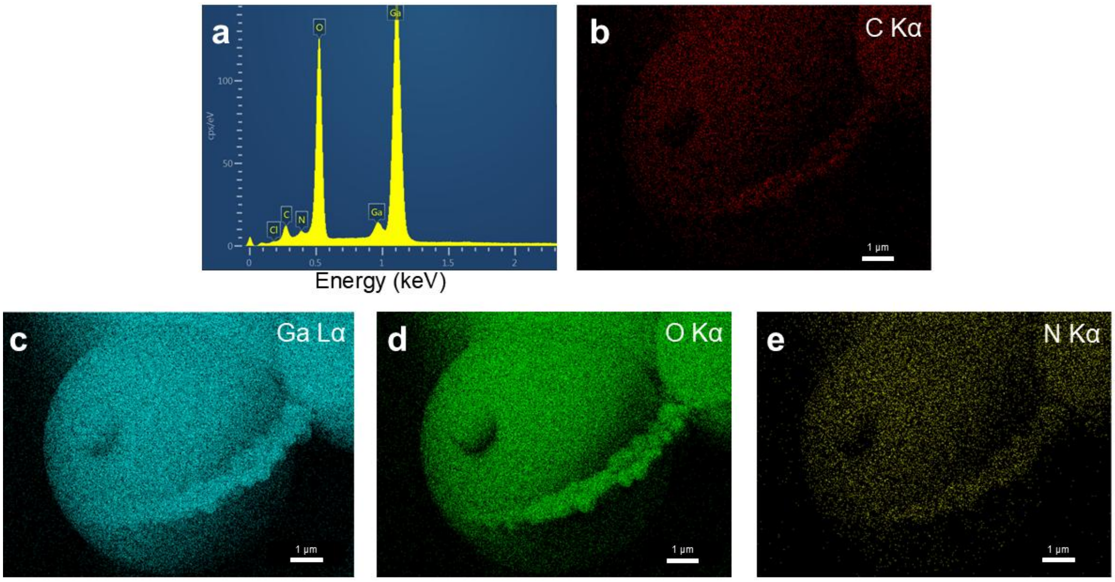

Figure S35. EDS results of the surface film on Ga after heat treatment at 450 °C using direct-contact NH_4Cl as nitrogen source. (a) Corresponding EDS spectrum, showing the sample dominated by Ga signals alongside O, N, and minor C signals. (b−e) EDS elemental distribution maps of (b) C, (c) Ga, (d) O, and (e) N.

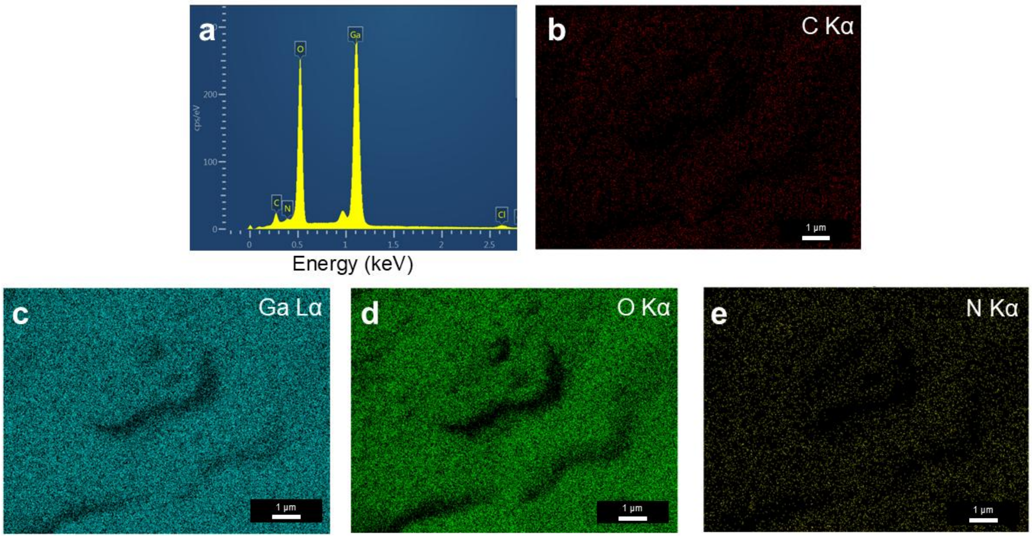

Figure S36. EDS results of the surface film on Ga after heat treatment at 450 °C using direct-contact NH_4Cl as nitrogen source. (a) Corresponding EDS spectrum, showing the sample dominated by Ga signals alongside O, N, and minor C signals. (b–e) EDS elemental distribution maps of (b) C, (c) Ga, (d) O, and (e) N.

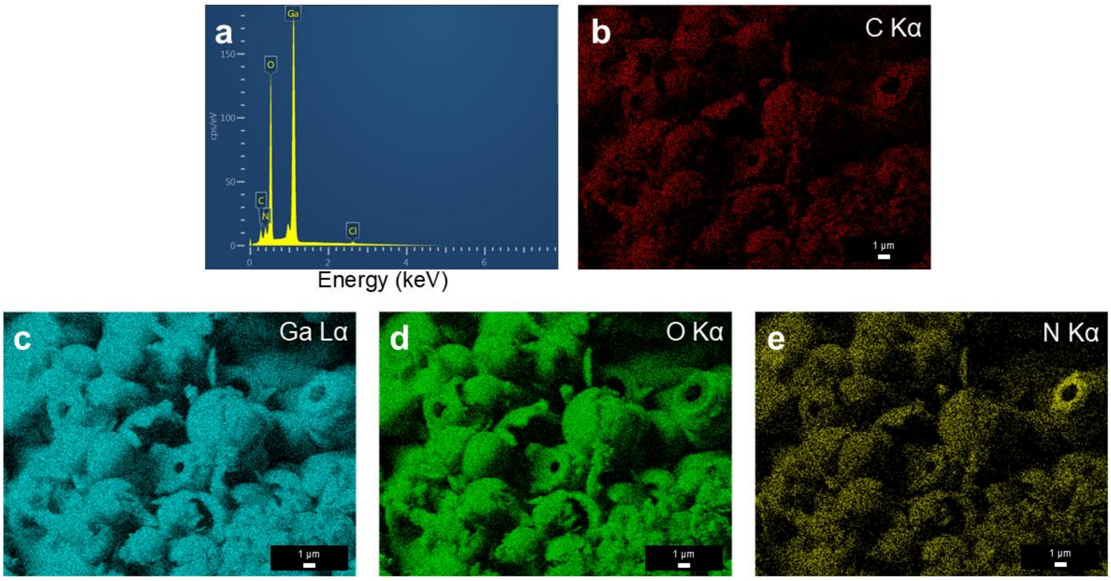

Figure S37. EDS results of the surface film on Ga after heat treatment at 500 °C using direct-contact NH_4Cl as nitrogen source. (a) Corresponding EDS spectrum, showing the sample dominated by Ga signals alongside O, N, and minor C signals. (b–e) EDS elemental distribution maps of (b) C, (c) Ga, (d) O, and (e) N.

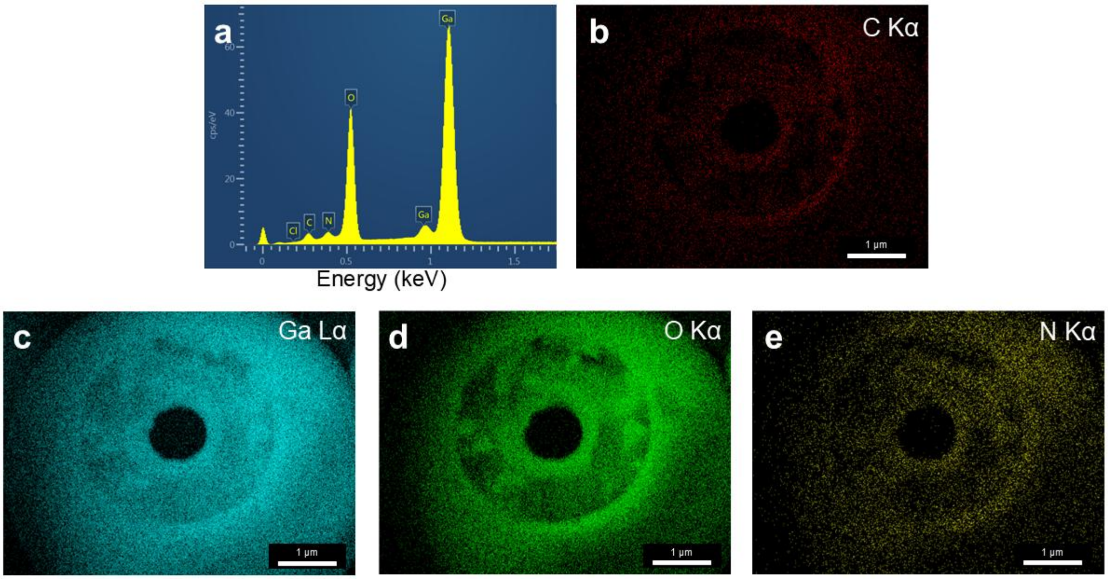

Figure S38. EDS results of the surface film on Ga after heat treatment at 500 °C using direct-contact NH_4Cl as nitrogen source. (a) Corresponding EDS spectrum, showing the sample dominated by Ga signals alongside O, N, and minor C signals. (b−e) EDS elemental distribution maps of (b) C, (c) Ga, (d) O, and (e) N.

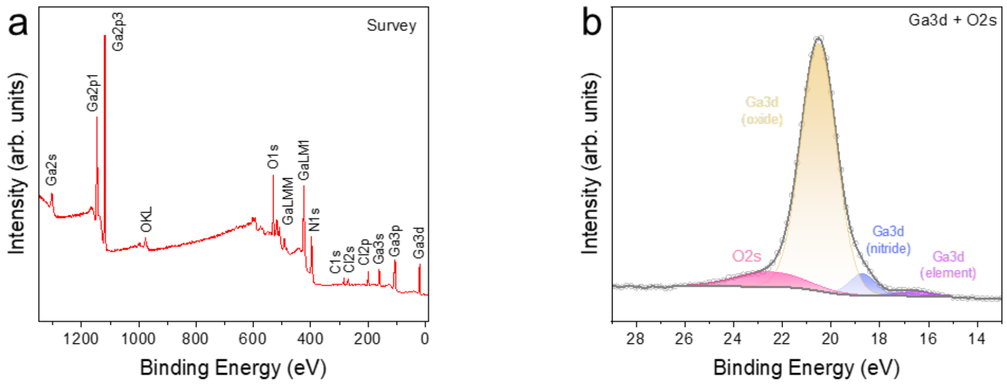

Figure S39. XPS results of the surface film formed on Ga after direct mixing of NH_4Cl with liquid Ga and heat treatment at 400 °C. (a) XPS survey spectrum. (b) High-resolution Ga 3*d* + O 2*s* spectrum with peak fitting.

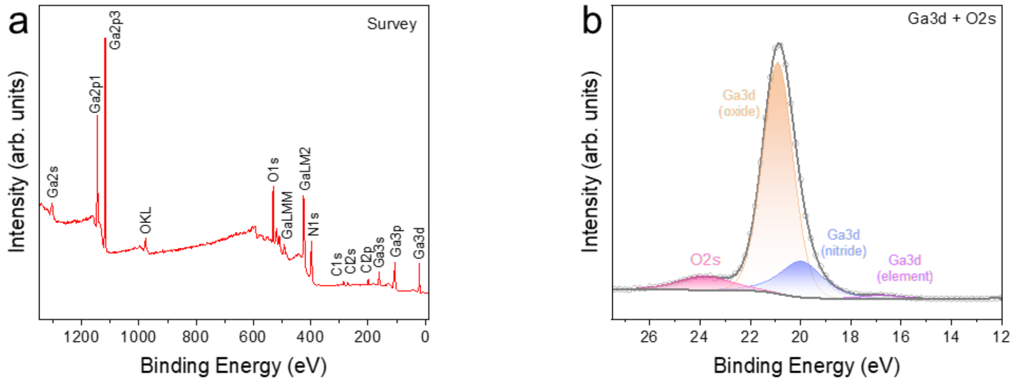

Figure S40. XPS results of the surface film formed on Ga after direct mixing of NH_4Cl with liquid Ga and heat treatment at 450 °C. (a) XPS survey spectrum. (b) High-resolution Ga 3*d* + O 2*s* spectrum with peak fitting.

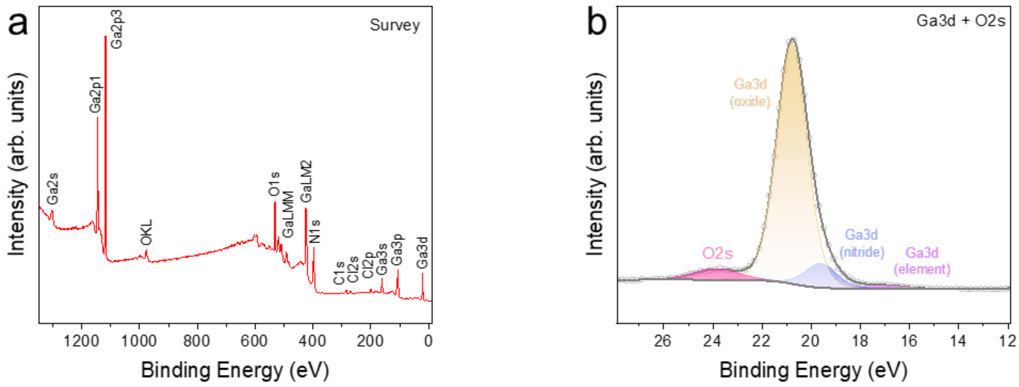

Figure S41. XPS results of the surface film formed on Ga after direct mixing of NH_4Cl with liquid Ga and heat treatment at 500 °C. (a) XPS survey spectrum. (b) High-resolution Ga 3*d* + O 2*s* spectrum with peak fitting.

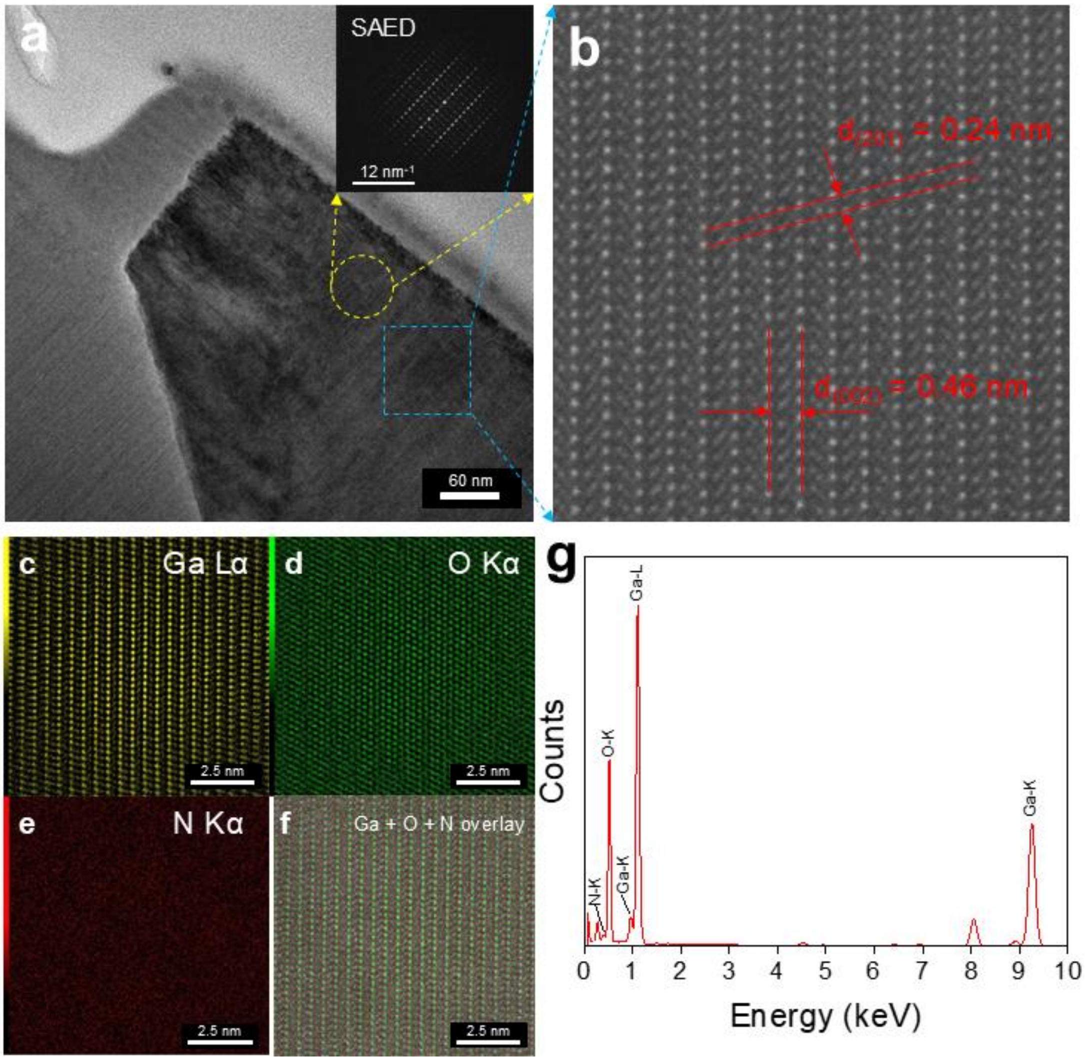

Figure S42. Aberration-corrected TEM and STEM-EDS results of a representative FIB-prepared microstructure obtained after heat treatment at 500 °C using NH_4Cl as a non-contact nitrogen source. (a) Low-magnification TEM image of the selected microstructure; the inset shows the corresponding selected-area electron diffraction (SAED) pattern, indicating local crystallinity. (b) Atomically resolved image showing lattice spacings of approximately 0.46 nm and 0.24 nm, which are consistent with the (002) and (201) planes of ε-Ga_2O_3, respectively. (c–e) STEM-EDS elemental maps of Ga, O, and N, respectively. (f) Overlay of the Ga, O, and N elemental maps. (g) Corresponding EDS spectrum. The peak near 8 keV is assigned to Cu Kα, which originates from the copper TEM grid. The Ga and O signals show clear spatial correspondence, whereas the N signal is much weaker than the Ga and O signals and does not show clear point-to-point correspondence with the observed lattice contrast. These results suggest that at least the analyzed local microstructure was dominated by a crystalline Ga–O-containing phase, likely ε-Ga_2O_3, with only weak nitrogen-related contribution.

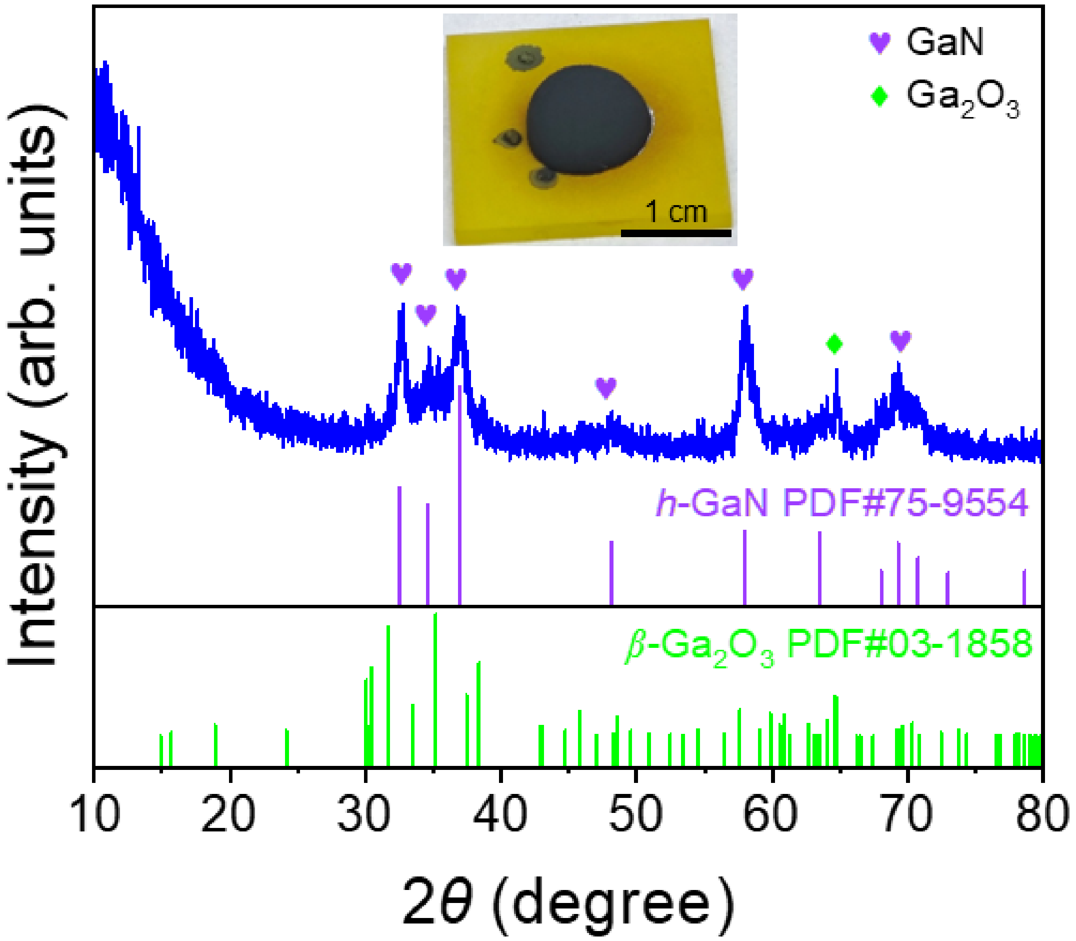

Figure S43. XRD pattern of liquid Ga surface film after heat treatment in N_2 at 800°C. Inset is a digital photograph of the post-experimental sample with the majority of liquid Ga underneath withdrawn on a quartz glass substrate. Several major reflections were consistent with *h*-GaN, while weak oxide-related reflections were also observed, indicating that nitridation became more evident under stronger thermal activation but oxidation remained a competing process.

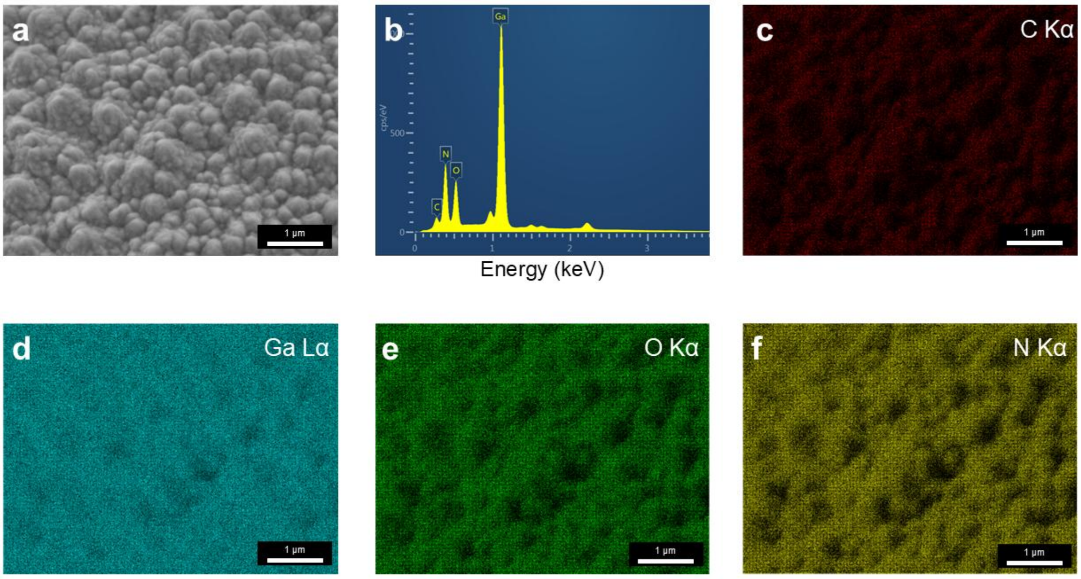

Figure S44. SEM and EDS results of the surface film on Ga after heat treatment at 800 °C in an N_2 atmosphere. (a) SEM image, (b) EDS spectrum acquired from the corresponding area, showing dominant Ga signals together with O and N signals and a minor C contribution. (c–f) EDS elemental maps of C (c), Ga (d), O (e) and N (f), respectively. The N signal is clearly detected in the product region, while O is also present, indicating the coexistence of nitrogen- and oxygen-containing species.

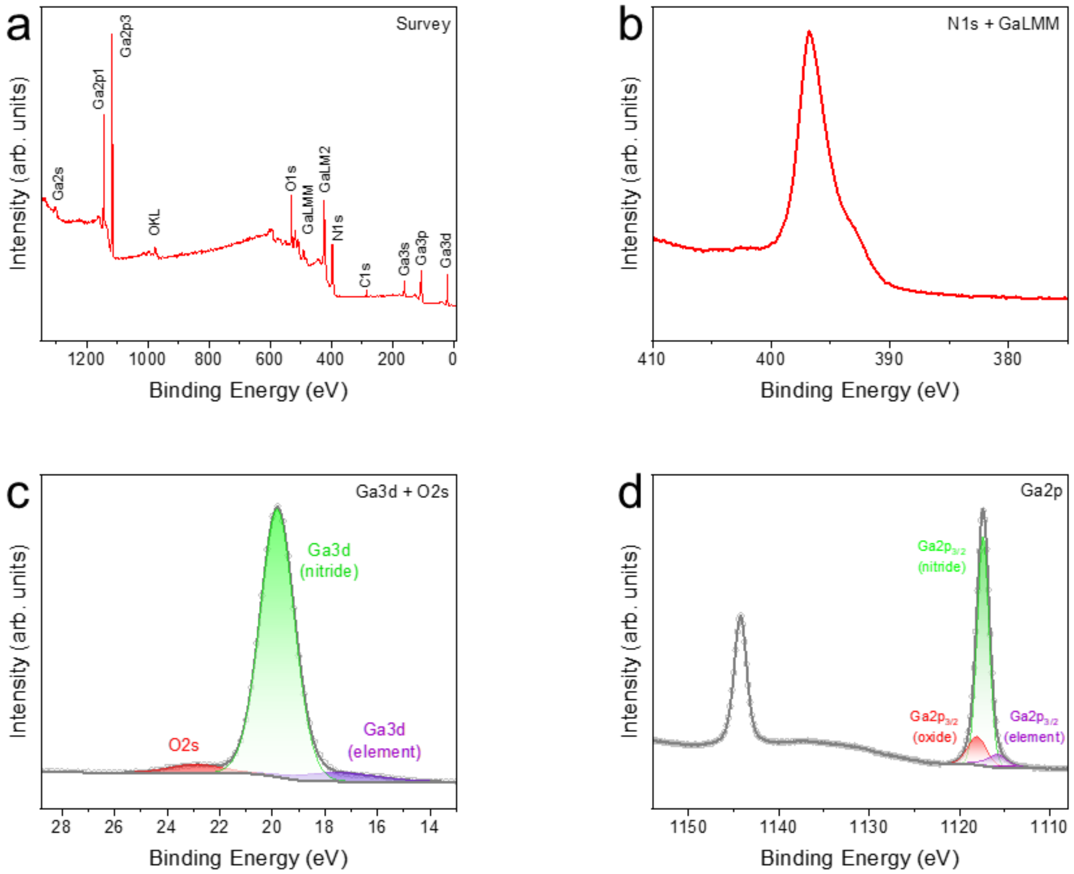

Figure S45. XPS results of the surface film on Ga after heat treatment at 800 °C in N_2. (a) XPS survey spectrum. (b) High-resolution N 1*s* + Ga LMM spectrum. (c) High-resolution Ga 3*d* + O 2*s* spectrum with peak fitting. (d) High-resolution Ga 2*p* spectrum with peak fitting. Although the N 1s region overlaps with the Ga LMM Auger feature, the spectral feature in this region, together with the Ga 3*d*/Ga 2*p* fitting results and XRD evidence, supports the formation of Ga–N-containing species at 800 °C.

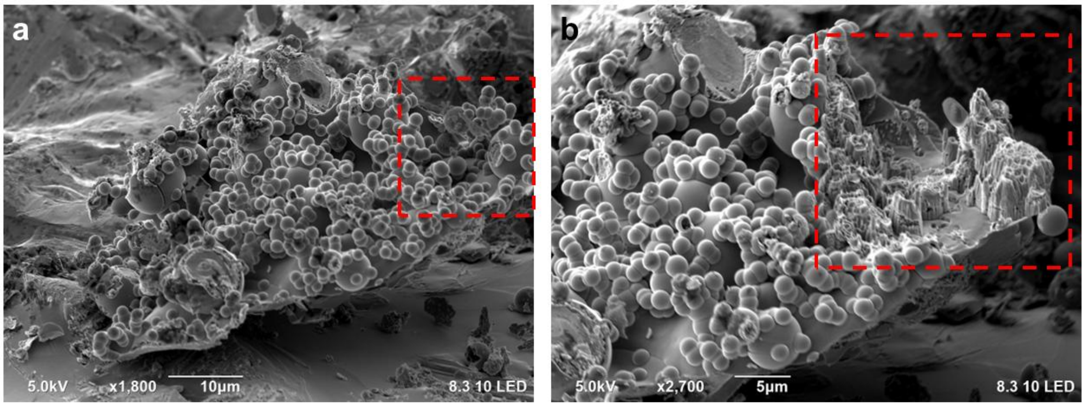

Figure S46. SEM images of the 500 °C direct-contact NH_4Cl sample recorded during FIB sample preparation. (a) SEM image before ion-beam focusing. (b) SEM image after ion-beam focusing. The red dashed boxes indicate the region used for ion-beam focusing. The focusing step was performed using an ion-beam accelerating voltage of 30 keV and a high-current setting of 30000 pA (30 nA). A pronounced morphological change was observed in the focused region after ion-beam exposure, indicating that the loosely aggregated/shell-like products were sensitive to high-current ion-beam irradiation during FIB operation. Therefore, FIB-based preparation of representative cross-sectional TEM specimens from this sample was not pursued further.
